# Probable basal allosauroid from the early Middle Jurassic Cañadón Asfalto Formation of Argentina highlights phylogenetic uncertainty in tetanuran theropod dinosaurs

**DOI:** 10.1038/s41598-019-53672-7

**Published:** 2019-12-11

**Authors:** Oliver W. M. Rauhut, Diego Pol

**Affiliations:** 1 SNSB-Bayerische Staatssammlung für Paläontologie und Geologie, Richard-Wagner-Str. 10, 80333 Munich, Germany; 2 Section Palaeontology & Geobiology, Department of Earth and Environmental Sciences, Ludwig-Maximilians-Universität, Richard-Wagner-Str. 10, 80333 Munich, Germany; 3 GeoBioCenter, Ludwig-Maximilians-Universität, Richard-Wagner-Str. 10, 80333 Munich, Germany; 4 CONICET, Museo Paleontológico Egidio Feruglio, Avenida Fontana 140, 9100 Trelew, Argentina

**Keywords:** Palaeontology, Evolutionary ecology

## Abstract

Tetanurae, the most successful clade of theropod dinosaurs, including modern birds, split into three major clades early in their evolutionary history: Megalosauroidea, Coelurosauria, and Allosauroidea. The oldest tetanurans occur in the earliest Middle Jurassic, but the early fossil record of the clade is still poor. Here we report one of the oldest known and most complete pre-Late Jurassic tetanuran, the probable allosauroid *Asfaltovenator vialidadi* gen. et sp. nov., which has an unusual character combination, uniting features currently considered to be apomorphic of different tetanuran lineages. A phylogenetic analysis resulted in a monophyletic Carnosauria (Allosauroidea + Megalosauroidea), and the inclusion of the new taxon significantly changes topology within carnosaurs. The analysis shows concentrated homoplasy in proximal nodes at the base of Tetanurae, and a temporal peak at the Pliensbachian-Toarcian extinction event, recently identified as a potential driver of tetanuran radiation. These results highlight the complex morphological evolution in the early radiation of tetanuran theropods, in which convergences and parallelisms were extremely common. This pattern seems to be a common feature in rapid radiation events of major clades of vertebrates and might explain the common difficulties to unravel phylogenetic relationships of important lineages at the base of major clades.

## Introduction

The Tetanurae represent the most diverse clade of theropod dinosaurs, which includes not only most of the well-known Mesozoic theropods, such as *Allosaurus* or *Tyrannosaurus*, but also modern birds^1^. The oldest tetanurans occur in the earliest Middle Jurassic^2–4^, but most of the known Middle Jurassic fossils are extremely fragmentary. Current phylogenetic analyses of tetanurans usually recover three principal lineages, the Megalosauroidea, Allosauroidea and Coelurosauria, but the exact relationships between these clades and the placement of many individual taxa remains controversial^2,4^. Recent research indicates an explosive radiation of tetanurans in the latest Early to Middle Jurassic^4^, but the fragmentary nature of the earliest known members of this clade hampers our understanding of this event. Here we report on a new basal tetanuran, *Asfaltovenator vialidadi* gen. et sp. nov., represented by the most complete skeleton known from early tetanuran history, which probably represents the oldest known representative of one of the main lineages, the Allosauroidea. The new taxon shows an unusual mosaic of tetanuran characters and highlights the high amount of convergences in the early evolution of the group and thus the resulting phylogenetic uncertainty in its early evolution. As this seems to be a common pattern in rapid radiations of major clades of vertebrates^5–8^, we present a quantitative analysis of homoplasy distribution both in the phylogenetic tree as well as in stratigraphic time to illuminate possible reasons for this observed pattern.

## Systematic Palaeontology

Theropoda Marsh, 1881, Tetanurae Gauthier, 1986, Allosauroidea (Marsh, 1878), *Asfaltovenator vialidadi* gen. et sp. nov.

## Etymology

Generic name for the Cañadón Asfalto Formation and *venator*, Greek for hunter. The species epithet honours the Administración de Vialidad Provincial of Chubut and the Dirección Nacional de Vialidad, for their aid to paleontological expeditions of the Museo Paleontológico Egidio Feruglio.

## Holotype

**Figure 1:**
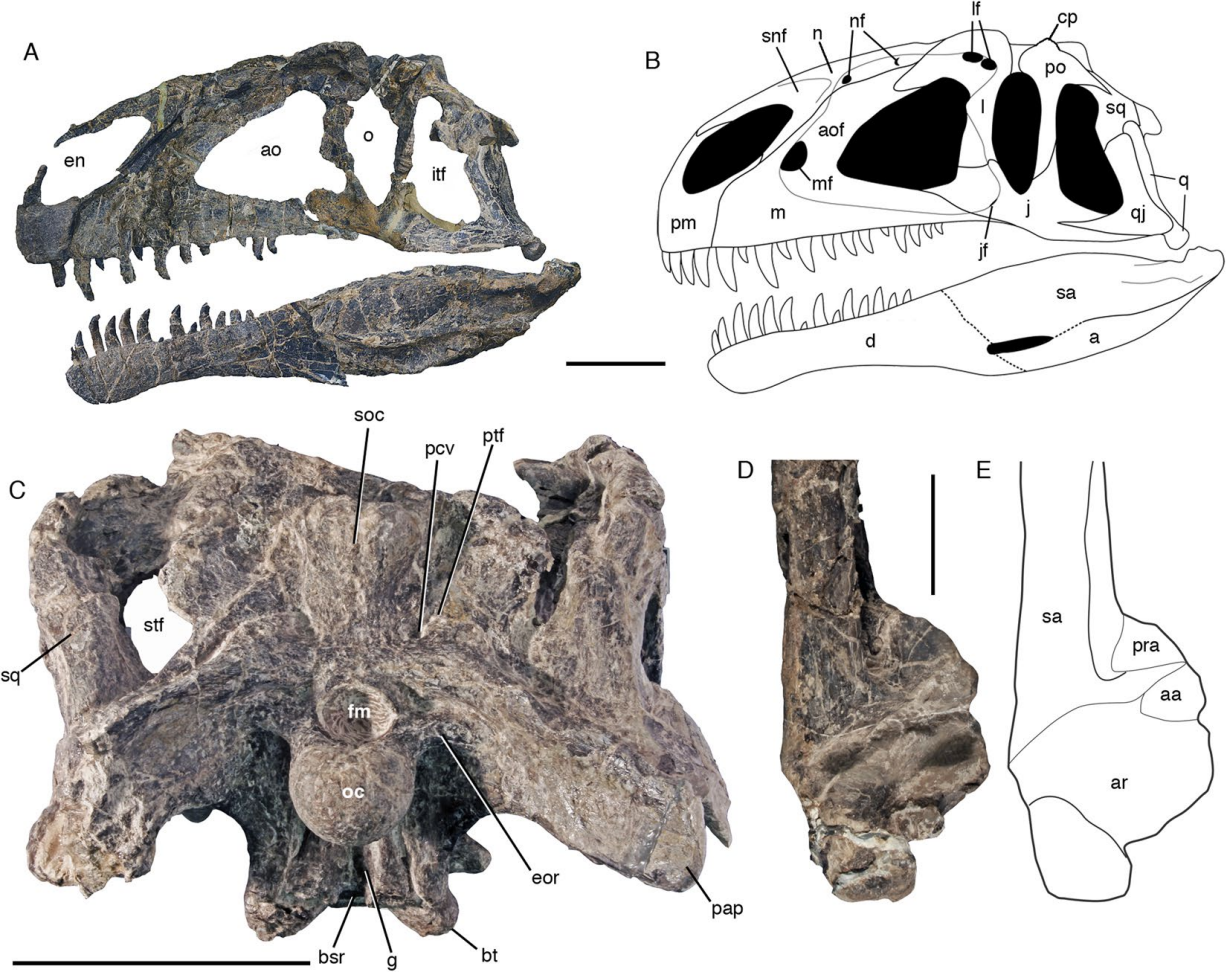
Cranial anatomy of *Asfaltovenator vialidadi*, MPEF PV 3440. (**A**) composite reconstruction of the skull and lower jaws, based on disarticulated cranial elements. (**B**), graphic reconstruction of articulated skull. (**C**), braincase in occipital view. (**D,E**) posterior end of left mandible in dorsal view; (**D**) photo; (**E**) outline drawing. Abbreviations: a, angular; aa, antarticular; ao, antorbital fenestra; aof, antorbital fossa; ar, articular; bsr, basisphenoid recess; bt, basal tubera; cp, cornual process; d, dentary; en, external nares; eor, exoccipital ridge; fm, foramen magnum; g, groove; itf, infratemporal fenestra; j, jugal; jf, jugal foramen; l, lacrimal; lf, lacrimal fenestrae; m, maxilla; mf, maxillary fenestra; n, nasal; nf, nasal foramina; o, orbit; oc, occipital condyle; pap, paroccipital process; pcf, posterior exit of mid-cerebral vein; pm, premaxilla; po, postorbital; pra, prearticular; ptf, posttemporal foramen; q, quadrate; qj, quadratojugal; sa, surangular; snf, supranarial fossa; soc, supraoccipital; sq, squamosal; stf, supratemporal fenestra. Scale bars are 10 cm (**A–C**) and 5 cm (**D**,**E**).

**Figure 2:**
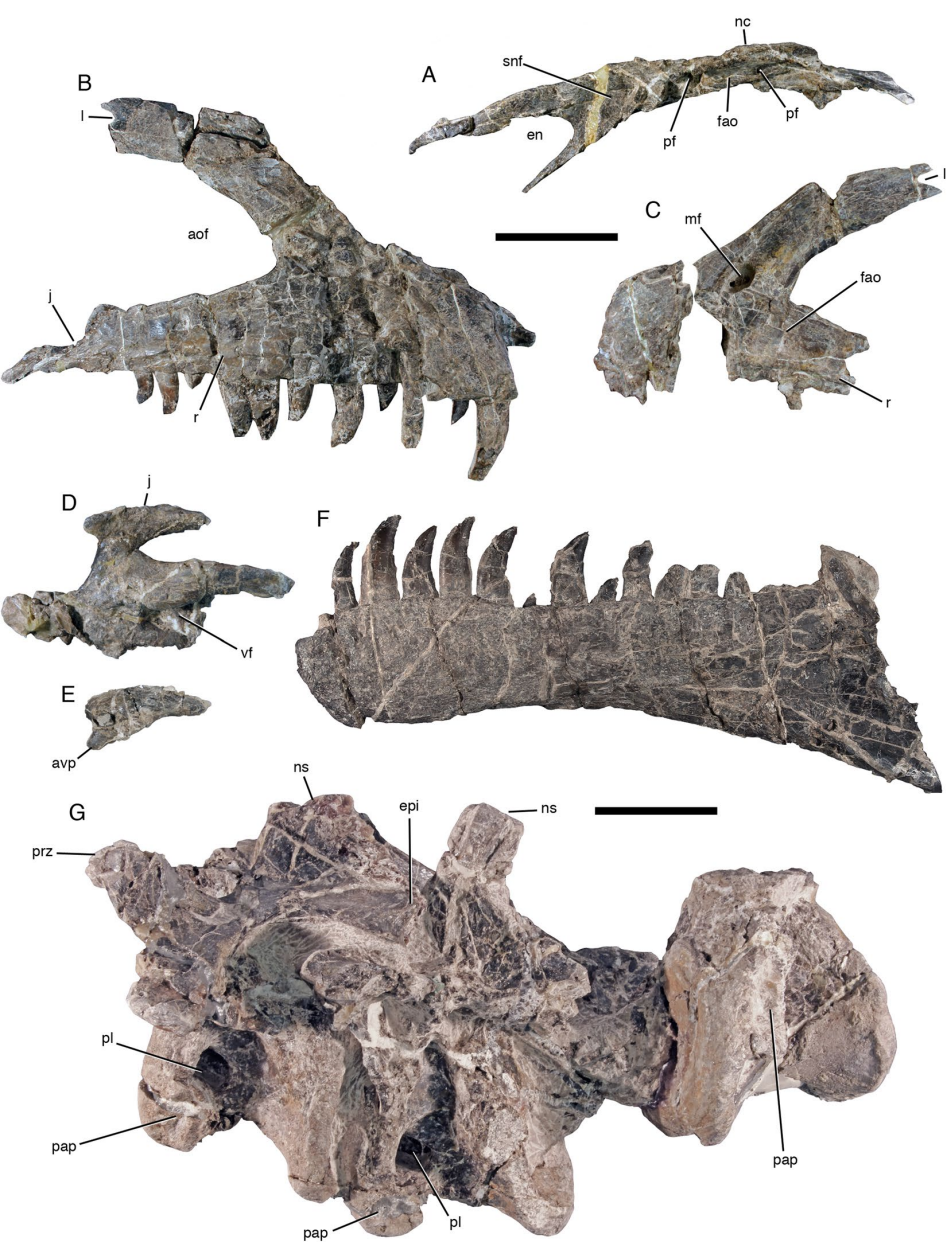
Selected skeletal elements of *Asfaltovenator vialidadi*, MPEF PV 3440. (**A**) left nasal in lateral view. (**B**), right maxilla in lateral view. (**C**) anterior end of left maxilla in lateral view. (**D**) left ectopterygoid in ventral view. (**E**) jugal process of left ectopterygoid in lateral view. (**F**) left dentary in lateral view. (**G**) last two cervical vertebrae and centrum of first dorsal vertebra in left lateral view. Abbreviations: aof, antorbital fenestra; avp, anteroventral process; en, external nares; epi, epipophysis; fao, antorbital fossa; j, facet for articulation with the jugal; l, facet for articulation with lacrimal; mf, maxillary fenestra; nc, nasal crest; ns, neural spine; pap, parapophysis; pf, pneumatic foramen; pl, pleurocoel; prz, prezygapophysis; r, ridge that forms the ventral border of the antorbital fossa; snf, supranarial fossa; vf, ventral fossa. Scale bars are 5 cm.

**Figure 3:**
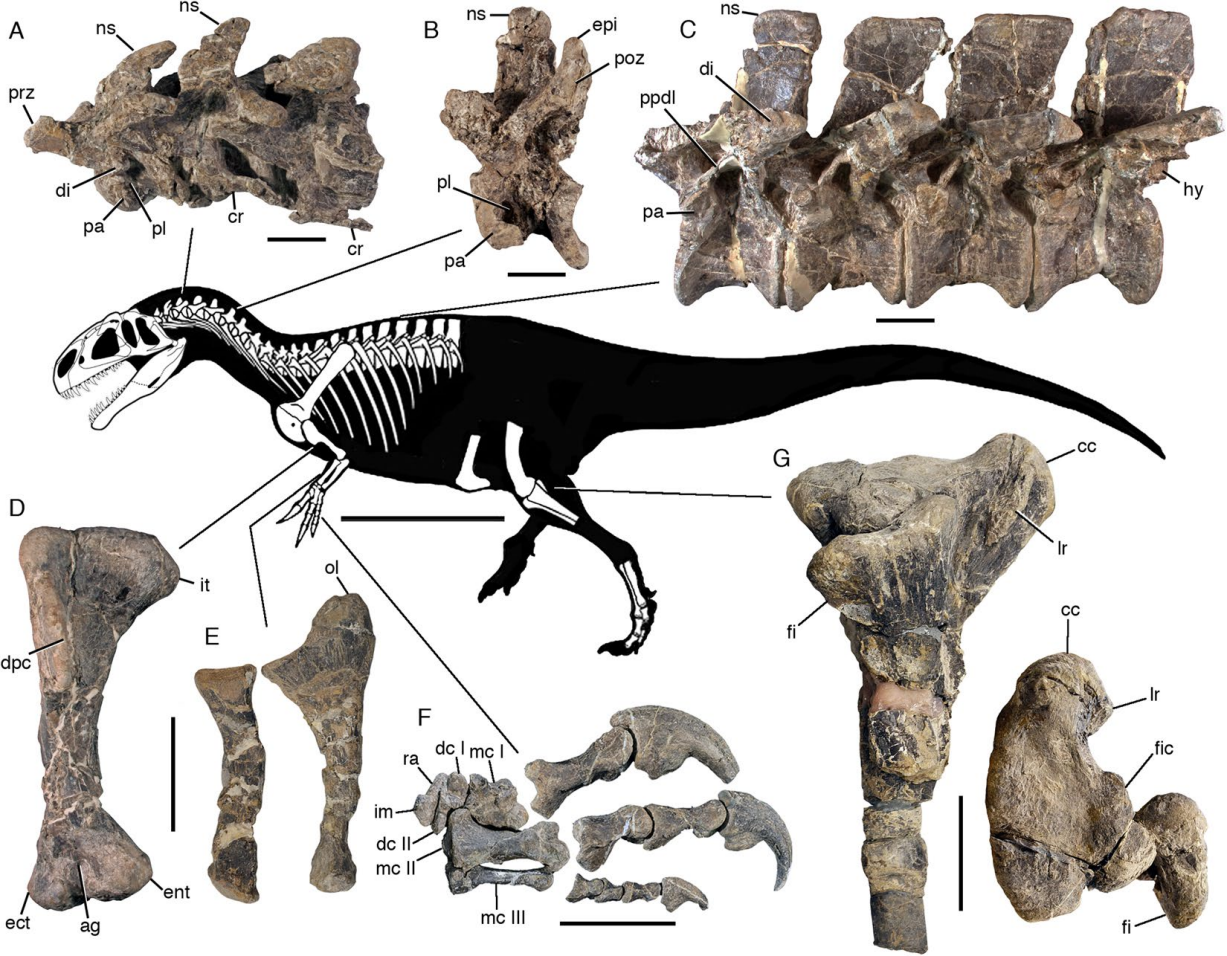
Skeletal reconstruction and postcranial anatomy of *Asfaltovenator vialidadi*, MPEF PV 3440. Centre: body outline with preserved elements indicated. (**A**) articulated cervical vertebrae three to five. (**B**) cervical vertebra 7. (**C**) articulated dorsal vertebrae four to seven (better preserved right side, reversed). (**D**) right humerus in anterior view. (**E**) right radius and ulna, medial view. (**F**) right manus, metacarpus in dorsal and digits in lateral view. (**G**) articulated proximal ends of right tibia and fibula in lateral and proximal views. Abbreviations: ag, anterior groove; cc, cnemial crest; cr, cervical rib; dc, distal carpal; di, diapophysis; dpc, deltpectoral crest; ec, ectepicondyle; ent, entepicondyle; epi, epipophysis; fi, fibula; fic, fibular condyle; hy, hyposphene; im, intermedium; it, internal tuberosity; lr, lateral ridge; mc, metacarpal; ns, neural spine; ol, olecranon; pa, parapophysis; pl, pleurocoel; poz, postzygapophysis; ppdl, paradiapophyseal lamina; prz, prezygapophysis; ra, radial. Scale bars are 100 cm (skeletal reconstruction), 5 cm (**A–C**) and 10 cm (**D–G**).

MPEF PV 3440, almost complete skull and partial skeleton (Figs 1–3).

## Locality and Horizon

Ca. 1.6 km NE of the village of Cerro Cóndor, lacustrine layers of the Cañadón Asfalto Formation, late Toarcian to Bajocian^9^.

## Diagnosis

Large basal tetanuran diagnosed by the following character combination (autapomorphies are marked with*): premaxillary teeth with well-developed distal, but only minute mesial serrations*; postorbital with small cornual dorsal process; exoccipital with pronounced horizontal ridges between paroccipital processes and foramen magnum*; ossified antarticular in the mandible; platycoelous cervical vertebrae; neural spines of cervical vertebrae three and four triangular and backswept*; anterior cervical epipophyses tab-like and elongated; mid-cervical vertebrae with median pit between parapophyses ventrally; ventral keel absent in posterior cervical and poorly developed in anterior dorsal vertebrae; well-developed paradiapohyseal lamina in middle and posterior dorsal vertebrae; dorsals 11 and 12 with small additional anterior centrodiapophyseal lamina*; articulated metacarpus broader than long; manual digit III significantly more slender and shorter than digits II and III.

## Description and Comparisons

*Asfaltovenator* is a large theropod, comparable in size to the well-known *Allosaurus*. The skull is 75–80 cm long, and the estimated body length of the holotype is 7–8 m. The reconstructed skull is high and slightly arched (Fig. 1A,B), similar to that of other allosauroids^2^. The premaxilla has an almost quadrangular body and a steeply arising anterior nasal process. In medial view, a large, posterodorsally opening foramen is present just below the narial margin, slightly posterior to its anterior end. The bone bears four teeth, with the anteriormost tooth being considerably smaller than the remaining premaxillary teeth. The maxilla has a short and high anterior ramus and an ascending process with a kinked anterior margin (Fig. 2B,C), as in many megalosaurids^2^. A large maxillary antorbital fossa is present and almost reaches the alveolar margin in the jugal ramus (Fig. 2B). Towards the anterior end of the antorbital fossa, at the base of the ascending process, there are a promaxillary foramen and a medially closed maxillary fenestra (Fig. 2C), similar to the situation in many megalosaurids^10^. The maxilla lacks a pneumatic recess on the medial side of the base of the ascending process. There were 13 maxillary teeth, which were held in place by separated interdental plates medially. The nasal has a large, sharply defined supranarial fossa and two pneumatic foramina within an expansion of the antorbital fossa onto its ventrolateral side (Fig. 2A), as in allosauroids. Similar to the situation in *Allosaurus*, the lateral margins of the nasals are raised into a crest that is continuous with a similar crest on the lacrimal. The lacrimal has a moderately developed lacrimal horn and a subdivided lacrimal fenestra, as in allosauroids^2^. The anterior and ventral rami of the lacrimal are of subequal length. The jugal expands anteriorly, as in most basal tetanurans, and the posterior, quadratojugal process is notably high at its base, higher than the jugal body below the orbit. A small pneumatic recess is present in the rim of the antorbital fossa in the anterior part of the bone. The frontal is notably robust, and the parietals are fused without visible suture. The postorbital is T-shaped, with a robust ‘brow’ in the supraorbital ramus and a small, triangular cornual process over the central body of the bone. The jugal articulation of the long, slender, but transversely robust jugal process forms a large, posteriorly opening trough, as in many megalosaurids. The supratemporal fossa does not extend onto the posterior process of the postorbital, in contrast to the situation in megalosaurids^2^, and, as in allosauroids, the dorsal lamina of the squamosal is continuous between the lateral and medial ramus, not invaginated by the supratmeporal fenestra. On the occiput (Fig. 1C), the posterior exit of the mid cerebral vein is connected to the posttemporal foramen by a curved depression, as in *Allosaurus*^11^, and the supraoccipital crest is rather low, but broad. The paroccipital processes are strongly ventrolaterally directed, with their tips lying entirely ventral to the foramen magnum, as in *Allosaurus*^11^. A broad vertical groove is present below the occipital condyle, as in most megalosauroids, but well-developed subcondylar recesses are absent, unlike the condition in *Piatnitzkysaurus*^12^ and *Eustreptospondylus*^13^. The basisphenoid has a deep basisphenoid recess and stout, dorsoventrally expanded and mainly anteriorly directed basipterygoid processes, similar to the condition in *Allosaurus*^11^. The palatine is tetraradiate with an expanded jugal process and a pneumatic recess dorsally. A pneumatic recess invaginates the ectopterygoid from medially (Fig. 2D), as in *Allosaurus* and other basal tetanurans. The jugal process of the ectopterygoid has a stout anteroventral process (Fig. 2E), as in *Dubreuillosaurus*^14^. A marked depression is present on the dorsal surface of the ectopterygoid wing of the pterygoid.

The dentary bears 14 teeth. It has a slightly expanded anterior end (Fig. 2F), as in most megalosauroids, and two meckelian foramina at the anterior end of the meckelian groove medially. As in the maxilla, the interdental plates are separate. The splenial has a forked posterior end and completely encloses a myliohyoid foramen. The mandibular fenestra was obviously small and might have been absent altogether, so that the anterior end of the surangular is high and accounts for more than half the height of the mandible at this level. A broad dorsal shelf is found on the posterior two-thirds of the surangular, bound medially by a raised medial flange, but the anterior border of the glenoid, which is formed by the surangular, is not notably raised, in contrast to most large basal tetanurans. A stout, triangular antarticular is present between the prearticular and the articular (Fig. 1D), a bone that was hitherto considered to be an autapomorphic neomorph of *Allosaurus*^11^. The retroarticular process is represented by a short, semioval, laterally placed posterior process of the articular that is broader than long. The articular surface for the *m. depressor mandibular* is developed as a broad, posterodorsally directed depression on the process.

There are ten cervical and 13 dorsal vertebrae. Cervical vertebrae are short and stout, with flat anterior and concave posterior articular surfaces (Figs 2G, 3A,B). Single pneumatic foramina are present on the anterolateral side in postaxial cervicals and anterior dorsals, but are absent in the axis and posterior dorsals. A very weak ventral ridge is present in the axis, but ventral keels are absent in postaxial cervicals and are poorly developed in dorsals one (Fig. 2G) and two, in contrast to *Piatnitzkysaurus* and *Condorraptor*, which have deep ventral keels in posterior cervical and anterior dorsal vertebrae^15,16^. The mid-cervical vertebrae have a shallow depression on the anterior part of the ventral side. Cervical neural arches have well-developed lateral lamination, large, laterally placed prezygapophyses and large, posterodorsolaterally directed epipophyses, which considerably overhang the postzygapophyses in anterior and mid-cervicals. The third cervical has an unusual, anteroposteriorly reduced neural spine that is strongly inclined posteriorly and has a curved anterior and thickened posterior margin (Fig. 3A). The spine of the fourth cervical has a straight ventral portion with parallel margins and a posteriorly inclined dorsal half with a slightly curved anterior margin. More posterior cervical neural spines are straight, with subparallel anterior and posterior margins and are taller than long. Dorsal vertebrae have spool-shaped, strongly constricted centra that lack pleurocoels or pronounced pleurocentral grooves. Lateral neural arch lamination is well developed, and neural spines are moderately high and rectangular (Fig. 3C). A small additional lamina is present in dorsals 11 and 12 between the paradiapophyseal lamina and the posterior centrodiapophyseal lamina. The anterior end of the hyposphene articular facet is marked by a notable step, as in *Piatnitzksaurus*^15^ and *Condorraptor*^16^. The last dorsal vertebra has anterolaterally directed transverse processes and is fused to the first sacral. The first sacral has an oblique ridge on the transverse process dorsally, as in *Condorraptor*^16^.

The furcula is not preserved, but both scapulocoracoids and forelimbs are complete. The scapula is notably broad, its length being approximately six times the minimal height of the shaft, but seems to be only slightly expanded distally, similar to the condition in megalosaurids. The acromion process is considerably, but more gradually expanded than in *Allosaurus*^11^, and a well-developed supraglenoid fossa is present on the proximal part of the scapula. The coracoid is semioval, higher than long, and has a well-developed, tapering posteroventral process. The biceps tubercle is developed as a ridge extending obliquely from the level of the dorsal margin of the glenoid anteroventrally, as in *Piatnitzkysaurus*^15^ and allosauroids. The humerus is robust and almost straight, as in several megalosauroids, and thus lacks the pronounced sigmoidal curve seen in *Piatnitzkysaurus*^15^ or *Allosaurus*^11^. The greater tubercle of the proximal humerus is robust and pronouced, though not to the degree seen in *Acrocanthosaurus*^17^. The deltopectoral crest is well-developed, anteriorly directed and extends for almost half the length of the humerus. The bone has a fossa on the anterior side of the distal end (Fig. 3D), as in allosauroids, and the distal articular surface is slightly canted medially. The antebrachium is short and robust, the radius being approximately 60% of the length of the humerus. The ulna has a large, robust olecranon process (Fig. 3E). The carpus consists of two proximal and two unfused distal carpals. The manus has three digits, without a remnant of the fourth metacarpal (Fig. 3F). The metacarpus is notably broad and stout, the articulated metacarpals being wider than long. Metacarpal I is slightly more than half the length of metacarpal II, closely appressed to the latter and very robust, whereas metacarpal III is very slender. The digits are rather short and massive, with digit I being the most robust and digit III being significantly more slender and shorter than the other digits.

Only the distal ends of the articulated pubes are preserved of the pelvic girdle. The pubic boot is moderately developed and only posteriorly expanded, with the distal ends of the left and right pubic boots being fused. The distal two thirds of the right femur are preserved. In contrast to many theropods, there is no depression for the attachment of *m. femorotibialis* on the anterior side of the distal end, but a well-developed extensor groove is present and extends onto the anterior side of the femur. Distally, the well-rounded condyles are separated by a broad, anteroposteriorly extending groove. The tibia has a well-developed, anteroproximally directed cnemial crest with rectangular outline (Fig. 3G). The fibular condyle is well offset from the cnemial crest by a broad incisura tibialis and does not reach as far posteriorly as the medial part. The fibular crest of the tibia is separated from the proximal end and thin, unlike the bulbous crest seen in some megalosaurids^10^. The proximal articular surface of the fibula is elongate kidney-shaped, being wider anteriorly than posteriorly and the bone lacks a pronounced groove on the medial side of the proximal end.

A poorly preserved right foot is present, including distal tarsal IV, metatarsals II to IV and several pedal phalanges. Metatarsals IV has a rounded anterolateral part of the proximal articular surface and a long posteromedial process, as in other basal tetanurans.

## Implications for Early Tetanuran Phylogeny

Recent phylogenetic analyses of basal tetanurans usually found three major lineages, the Megalosauroidea, Allosauroidea and Coelurosauria, with a sister-group relationship between allosauroids and coelurosaurs, to the exclusion of megalosauroids^2,4,10,14,18–21^. *Asfaltovenator* shows an unusual character combination, combining features previously considered synapomorphic of Megalosauroidea with supposed synapomorphies of Allosauroidea and characters that are plesiomorphic for tetanurans in general. Megalosauroid characters include a pronounced kink in the anterodorsal margin of the maxillary ascending process, a medially closed maxillary fenestra, a deep posterior groove on ventral process of postorbital, a broad fossa below the occipital condyle, an anterior expansion of the dentary, the presence of a ridge delimitating the anterior margin of the hyposphene articular facet, and the absence of a proximal medial groove in the fibula^2^. Allosauroid features include the presence of a pronounced supranarial fossa, the nasal participation in the antorbital fossa, presence of pneumatic foramina in the nasal, presence of lateral nasal crests, a subdivided lacrimal fenestra, a continuous, uninvaginated dorsal lamina of the squamosal, the strongly ventrolaterally directed paroccipital processes, the ridge-like biceps tubercle on the coracoid, and the presence of a distal humeral fossa^2^. Plesiomorphic characters include the absence of a medial pneumatic recess in the maxilla, flat anterior articular surfaces of cervical vertebrae, and the broad scapular blade. *Asfaltovenator* furthermore shows characters previously regarded as autapomorphies of different taxa, including the anteroventral process on the jugal ramus of the ectopterygoid (proposed autapomorphy of *Dubreuillosaurus*^14^), a low, rectangular and backswept neural spine in the third cervical vertebra (proposed autapomorphy of *Afrovenator*^22^), and an ossified antarticular (proposed autapomorphy of *Allosaurus*^11^).

**Figure 4:**
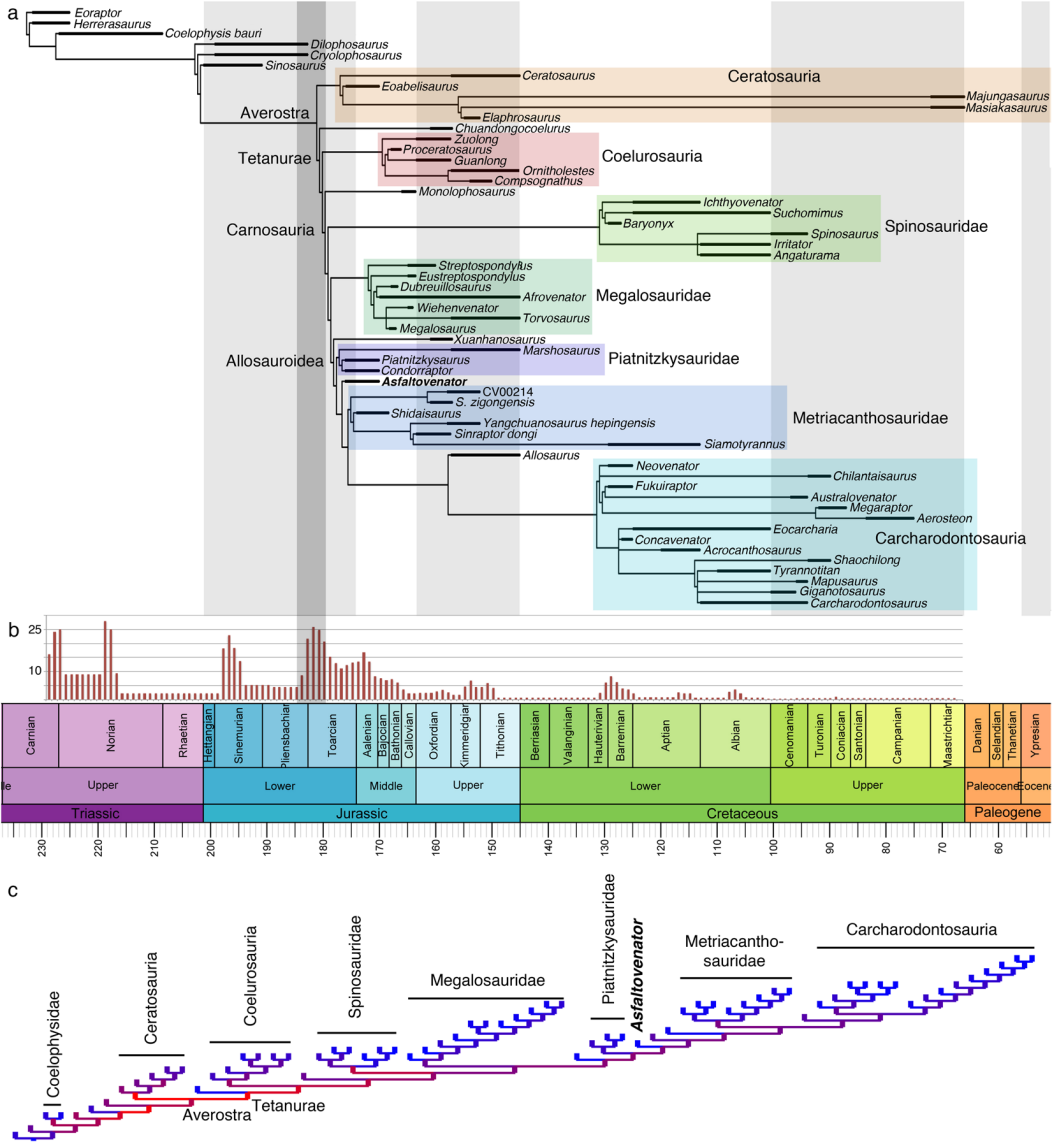
Phylogenetic position of *Asfaltovenator* and analysis of homoplasy distribution in tetanuran phylogeny. (**A**) time calibrated reduced consensus tree. (**B**) distribution of homoplasy over time in tetanuran evolution. Numbers refer to average number of homoplasies per one million year time bin. (**C**) colour-coded tree showing the concentration of homoplasy at the base of Tetanurae (red; see Supplementary Information for details). The grey line in (**A**,**B**) indicates the Pliensbachian-Toarcian extinction event.

To test the phylogenetic affinities of *Asfaltovenator*, we included it in a modified version of the data matrix of one of the most recent analyses of basal tetanurans^4^ (see Supplementary Information). The analysis leads to considerable changes in basal tetanuran phylogeny (Fig. 4A). Not only are the taxa that constituted Megalosauroidea and Allosauroidea grouped in a monophyletic Carnosauria to the exclusion of Coelurosauria, but several subclades of Megalosauroidea are found as consecutive outgroups to a monophyletic Allosauroidea. Thus, Spinosauridae are retrieved as the first major clade to branch off the lineage leading towards Allosauroidea, followed by Megalosauridae and Piatnitzkysauridae (Fig. 4A; see Supplementary Information).

A quantitative analysis of the distribution of homoplasy within stratigraphic time shows that the highest peak in homoplasy among tetanurans occurred during the late Pliensbachian and Toarcian by the end of the Early Jurassic (Fig. 4B). Looking at homoplasy concentration in the phylogenetic trees shows high levels and concentration of homoplastic changes in proximal nodes at the origin of Averostra and tetanurans, and among basal nodes of Tetanurae, which gradually diminish towards more derived nodes of this clade (Fig. 4C). This concentration of homoplasy during these rapid radiations is compatible with the increase in general rates of morphological change during this time inferred by previous authors^23,24^ (see also Supplementary Materials).

The discovery of *Asfaltovenator*, one of the oldest and most complete pre-Late Jurassic tetanuran theropods known^3,4^, thus highlights the uncertainty that still surrounds the relationships of the major lineages of the most diverse clade of theropod dinosaurs. The unusual character combination shown by this taxon alters the optimization of characters previously regarded as synapomorphies of major clades. However, alternative topologies, including a traditional Megalosauroidea are only slightly less parsimonious (see Supplementary Information). The abundance and concentration of parallelisms and convergences during the early radiation of the clade explains the difficulties in the establishment of interrelationships of major clades of Tetanurae. High levels of homoplasy and the parallel and mosaic-like occurrence of characters in different lineages of early tetanurans make secure inferences about the relationships of the major clades and their subordinate lineages currently impossible (Fig. 4B,C; Supplementary Information). However, later representatives of the different lineages can more readily be differentiated.

## Homoplasy and Rapid Radiation Events

Recently, the Toarcian Anoxic Event (TAE; also known as Pliensbachian-Toarcian extinction event) has come into focus as a possible important driver of Jurassic dinosaur evolution^25–27^. The TAE has been linked to massive volcanic eruptions of the Karoo-Ferrar igneous province^28,29^, leading to several pulses of extinctions in the late Pliensbachian and early Toarcian^30^. Although the effects of this Pliensbachian-Toarcian extinction event on marine invertebrates are rather well studied^30–35^, its impact on vertebrate evolution, and especially the evolution of terrestrial vertebrates, is still less well understood. However, it has recently been suggested that it led to an explosive radiation of tetanuran theropods in the Middle Jurassic^4^, and important radiations are also seen in other clades of terrestrial vertebrates during that time^25,36^. Interestingly, the temporal peak of morphological change (and homoplastic change) found here coincides with the TAE, with rates gradually decreasing during the Middle Jurassic (Fig. 4B). Increased rates of morphological evolution have been reported to follow extinction events like this one in other groups^37,38^. It has been suggested that species richness (taxonomic diversity) might primarily be related to available ecospace^39^, so the availability of the supercontinent Pangea after the TAE might account for the taxonomic success of tetanurans in their early evolution. Relaxed natural selection due to ecological release^40^ could lead to a marked increase in morphological rate of evolution that will also result in an increase in homoplasy, evidenced in closely related nodes during this rapid radiation. Increasing annidation and specialisation and thus stabilising selection in later stages of tetanuran evolution, when the available ecological niches are subsequently filled, likely fixed different traits in the phenotype and led to more clearly recognisable lineages. This is in accordance with the distribution of rates of morphological change and homoplasy seen both in tetanuran phylogeny and geological time, where concentration of morphological change (and homoplasy) decreases in branches more remote to the origin of Tetanurae and, more or less gradually, after the TAE (Fig. 4; see Supplementary Materials). Thus, these scnearios might explain the fit of phylogenetic inertia model to the distribution of homoplasies in phylogenies, as noted for several clades^41^. This pattern is probably not restricted to radiations following extinction events, but might act under other instances of ecological release, such as the exploration of new ecological regimes, either due to the invasion of a new habitat or the development of some key innovation^40^, and could thus potentially also explain high levels of morphological evolution (and homoplasy) often seen at the base of other major clades^5–8^. In consequence, high levels of homoplasy on proximal nodes make a reconstruction of the detailed relationships of lineages resulting from such radiation events difficult; this is especially the case if many basal members are only known from very incomplete material, as it is the case in tetanurans. Good sampling of basal members of the different clades and the availability of complete specimens will be key to resolve such problems.

## Materials and Methods

The phylogenetic position of the new taxon was tested using a phylogenetic data matrix including 66 taxa and 355 osteological characters, modified from the most recent analysis of basal tetanurans^4^. The data matrix was analysed with equally weighted parsimony using TNT, version 1.1^42^. The same matrix was used to analyse distribution of homoplasy both within the phylogeny and in time. For these analyses, one of us (DP) wrote a script for the software TNT that quantified the proximity of homoplasy steps on all branches of each the most parsimonious trees, as well as counting the number of homoplastic characters per one million year time bin on a time-calibrated phylogenetic tree. Homoplasy concentration (i.e., proximity of homoplasies) for a given branch was calculated as the sum over all characters of the sums of the inverse nodal distances of homoplastic transformations to the same character state divided by the total number of homoplastic convergences/parallelisms. For the time bin analysis, a time-calibrated phylogenetic tree was created using the stratigraphic ages of first appearance for each taxon. We then calculated the average number of homoplastic changes per one million year time bin over all branches crossing this time bin, also taking ghost lineages into account. The scripts for the analyses are included in the supplementary information. For further details on methodology see Supplementary Information.

## Supplementary information

Supplementary information

## Data Availability

The phylogenetic data is included in the spplementary information and the data matrix is available on morphobank (morphobank.org). The TNT script to calculate rates of homoplasy as well as the stratigraphic data are available as supplementary files.
