# Supplementary material for: Probable basal allosauroid from the early Middle Jurassic Cañadón Asfalto Formation of Argentina highlights phylogenetic uncertainty in tetanuran theropod dinosaurs
Source: Sci Rep. 2019 Dec 11;9:18826. doi: 10.1038/s41598-019-53672-7 (PMC6906444; doi:10.1038/s41598-019-53672-7)
Supplement: Supplementary file 1 — Supplementary information [file 41598_2019_53672_MOESM1_ESM.docx]

Supplementary Information for

Probable basal allosauroid from the early Middle Jurassic Cañadón Asfalto Formation of Argentina highlights phylogenetic uncertainty in tetanuran theropod dinosaurs

Oliver W. M. Rauhut, Diego Pol

Correspondence to: [rauhut@snsb.de](mailto:xxxxx@xxxx.xxx)

**This PDF file includes:**

Supplementary information on taxonomic distinctiveness of *Asfaltovenator*

Supplementary information on phylogenetic analysis, including character list

Additional information on methodology of homoplasy analysis

Figure S1. Strict consensus tree of phylogenetic analysis.

Figure S2. Bremer support and bootstrap values of nodes of phylogenetic analysis.

Figure S3. Bremer support and jackknive values for trees excluding particularly unstable taxa.

Figure S4. Strict and reduced consensus tree of phylogenetic analysis excluding *Asfaltovenator*.

Figure S5. Reduced consensus tree with labelled nodes.

Figure S6. Colour-coded homoplasy distribution mapped on the reduced consensus tree.

Table S1. Measurements of forelimb elements of *Asfaltovenator*.

Table S2. Ages assigned to taxa for time calibrated cladograms and analysis of homoplasy over time bins (same as FADLAD file).

References

**Other Supplementary Materials for this manuscript include the following:**

Supplementary file 1. TNT script “phomoplasy.run” for the analysis of homoplasy concentration on a calibrated tree, evolutionary rates and homoplasy distribution over geological time.

Supplementary file 2. FADLAD file with first appearance datum and last appearance datum for each terminal taxa (required by the script phomoplasy.run to calibrate the phylogenies)

External Database S1. Phylogenetic matrix (as external file on morphobank).

Distinction of *Asfaltovenator* from the contemporaneous *Piatnitzkysaurus* and *Condorraptor*

*Asfaltovenator* is the fourth medium to large sized theropod described from the Cañadón Asfalto Formation^16,27,43^, and the third basal tetanuran from this unit. Given that such a high diversity of basal tetanurans shortly after the first occurrence of the clade might be surprising, we offer some additional comparisons with the two previously known tetanurans from the Cañadón Asfalto Formation to support its distinctiveness.

*Asfaltovenator* differs from *Piatnitzkysaurus* in numerous characters^12,15,43^. In the cranial skeleton, these characters include the more steeply inclined ascending process of the maxilla, lack of an inflated base of the ascending process of the maxilla, maxillary tooth count (13 versus 18), the strongly developed lateral exoccipital ridges in the occiput, the wider ventral groove below the occipital condyle and less developed subcondylar recesses on the occiput, the dorsoventrally more expanded basipterygoid processes, and the more rounded outline and lack of a ventral process at the anterior end of the dentary. In the vertebral column, differences include the relatively shorter mid-cervical vertebrae, the absent or poorly developed ventral keels in the posteriormost cervicals and anterior dorsal vertebrae, and the additional centrodiapophyseal lamina in posterior dorsals. In the forelimb, the humerus of *Asfaltovenator* is notably more robust and lacks the sigmoidal curve seen in the humerus of *Piatnitzkysaurus*, and the ulnae of both animals are of approximately the same length, despite the holotype of *Asfaltovenator* representing a considerably larger animal than the material of *Piatnitzkysaurus*, indicating that the former had a relatively shorter antebrachium.

Comparisons with *Condorraptor* are more difficult due to the restricted material known for this taxon^16^. Nevertheless, important differences found in the presacral vertebrae include the relatively shorter mid-cervical vertebrae, the lack of a ventral keel in the last cervical and poorly developed ventral keels in the anterior dorsal vertebrae, the presence of an additional centrodiapophyseal lamina in posterior dorsals, the lack of a rounded expansion of the interprezygapophyseal slit in posterior dorsals and anterior sacrals, and the more strongly developed and slightly proximally inclined cnemial crest of the tibia in *Asfaltovenator*.

Given these marked differences between *Asfaltovenator* and the other two named taxa, as well as the differences between *Codorraptor* and *Piatnitzkysaurus* listed by Rauhut^16^ (p. 106), three basal tetanuran theropods were clearly present in the Cañadón Asfalto Formation.

Phylogenetic analysis

Analytical procedures

In order to test the phylogenetic position of the new taxon, we included it in a datamatrix of two outgroups and 66 theropod ingroup taxa, with emphasis on basal tetanuran theropods. To improve taxon and character sampling for both tetanuran outgroups and basal tetanurans, we added the largely complete, early Middle Jurassic ceratosaur *Eoabelisaurus*^27^ and the Jurassic coelurosaurs *Coelurus*^44,45^ and *Zuolong*^46^. The taxa were scored for 355 osteological characters. Most characters were taken from Rauhut et al.^4^, which, in turn, was based on Carrano et al.^2^, with some additions and changes, as outlined in the character list below. Thirty-two of the characters were treated as ordered, the rest as unordered. The data matrix can be found on Morphobank (morphobank.org) under project 3267. The matrix was analysed using the traditional search option in TNT^42^ using equally weighted parsimony, with 1000 replicates of Wagner trees, followed by TBR branch swapping.

Results

The analysis resulted in 95142 equally parsimonious trees (MPTs) with a length of 1324 steps (CI: 0.338; RI: 0.633; RC: 0.214). Additionally, in order to test the influence of *Asfaltovenator* on tetanuran interrelationships, we performed a second analysis of the same matrix and analytical procedures, just with the new taxon excluded (see below for results). This analysis resulted in 4500 equally parsimonious trees with a length of 1290 steps (CI: 0.385; RI: 0.635; RC: 0.244).

Notes on nomenclature used

As our analysis differs in several important aspects from other current analyses of theropods, we deem it useful to clarify our use of clade names. Thus, in the following, we use the following clade definitions:

Averostra: We follow the node-based definition of Ezcurra & Cuny^47^ of Averostra as the clade that includes *Ceratosaurus nasicornis* and *Allosaurus fragilis* and all descendants of their most recent common ancestors.

Ceratosauria: Following the stem-based definition of Padian et al.^48^, Ceratosauria is the clade including *Ceratosaurus nasicornis* but not Neornithes.

Tetanurae: As in Xu et al.^49^ and in accordance with Carrano et al.^2^, we define Tetanurae as a stem-based clade that includes Neornithes but not *Ceratosaurus nasicornis*.

Coelurosauria: In agreement with Sereno^50^, we consider Coelurosauria to be a stem-based clade defined as all theropods that are more closely related to Neornithes than to *Allosaurus fragilis*.

Carnosauria: Carnosauria is defined as a stem-based clade that includes all theropods that are more closely related to *Allosaurus fragilis* and to *Megalosaurus bucklandii* than to Neornithes. We thus emend the definition of Padian et al.^48^, which used only *Allosaurus fragilis* as specifier and is thus equivalent to the definition of Allosauroidea by Sereno^50^ and Brusatte & Sereno^51^. This emended definition more closely corresponds to the original concept of Carnosauria as proposed by Huene^52,53^, and is not applicable in phylogenetic hypotheses that do not find support for a megalosaurid-allosaurid clade. We therefore also use a more restricted definition of Allosauroidea (see below).

Megalosauroidea: Megalosauroidea is a stem-based clade including all theropods that are more closely related to *Megalosaurus bucklandii* than to either *Allosaurus fragilis* or *Tyrannosaurus rex*^2^.

Spinosauridae: Spinosauridae includes all theropods that are more closely related to *Spinosaurus aegyptiacus* than to either *Megalosaurus bucklandii* or *Allosaurus fragilis*.

Piatnitzkysauridae: As the position of Piatnitzkysauridae in our analysis differs from that proposed by Carrano et al.^2^ and would be equivalent to Allosauroidea in its taxonomic content if using the definition proposed by these authors, we deem it necessary to extend the phylogenetic definition of this clade. Thus, Piatnitzkysauridae includes all theropods that are more closely related to *Piatnitzkysaurus floresi* than to either *Spinosaurus aegyptiacus*, *Megalosaurus bucklandii* or *Allosaurus fragilis*.

Allosauroidea: In contrast to the node-based definition of Allosauroidea employed by Padian et al.^48^, who defined this clade as Sinraptoridae and Allosauridae and all descendants of their most recent common ancestor, we prefer a stem-based definition of this clade, as originally proposed by Sereno^50^, to better reflect the principle subdivision of tetanurans into three main lineages, Megalosauroidea, Coelurosauria and Allosauroidea, found in the vast majority of recent analyses. However, as the definition given by Sereno^50^ and Brusatte & Sereno^51^ would equal the taxonomic contents of the Carnosauria under the tree topology recovered here, we emend the definition as follows: Allosauroidea includes all theropods that are more closely related to *Allosaurus fragilis* than to either *Megalosaurus bucklandii* or Neornithes.

Metriacanthosauridae: Metriacanthosauridae was originally defined on the basis of differential characters by Paul^54^ and has recently been revived as a senior synonym for Sinraptoridae by Carrano et al.^2^, who, however, did not provide a phylogenetic definition. We use the name here for a stem-based clade that includes all theropods that are more closely related to *Metriacanthosaurus parkeri* than to either *Allosaurus fragilis*, *Carcharodontosaurus saharicus*, *Megalosaurus bucklandii*, or *Spinosaurus aegyptiacus*.

Allosauria: As with Metriacanthosauridae, Allosauria was originally proposed by Paul^54^, and recently revived by Carrano et al.^2^, although without providing a phylogenetic definition. We use Allosauria here for a node-based taxon including *Allosaurus fragilis* and *Carcharodontosaurus saharicus* and all descendants of their last recent common ancestor.

Allosauridae: As in Padian et al.^48^, we regard Allosauridae as a stem-based taxon, being defined as all theropods that are more closely related to *Allosaurus fragilis* than to either *Carcharodontosaurus saharicus* or *Sinraptor dongi*. Although *Metriacanthosaurus parkeri* is the specifier for the Metriacanthosauridae, the sister taxon to Allosauria in the current tree topology, we prefer to use the much better known *Sinraptor dongi*^55^ in our definition.

Carcharodontosauria: Carcharodontosauria was defined by Benson et al.^19^ to refer to the clade including Neovenatoridae and Carcharodontosauridae, in contrast to the stem-based definition of the latter family employed by Brusatte & Sereno^51^, which would make Neovenatoridae part of Carcharodontosauridae. We thus follow Benson et al.^19^ in their stem-based definition of Carcharodontosauria as the clade of all theropods that are more closely related to *Neovenator salerii* and *Carcharodontosaurus saharicus* than to either *Allosaurus fragilis* or *Sinraptor dongi*.

Consensus trees

*Strict consensus*. This tree (Fig. S1) shows a rather well-resolved backbone of general theropod relationships, including monophyletic Averostra, monophyletic Ceratosauria, and a monophyletic Tetanurae. Within Tetanurae, a sister-group relationship between a monophyletic Coelurosauria and a monophyletic Carnosauria is recovered. Within the latter clade, Spinosauridae form the outgroup to a monophyletic clade that includes Megalosauridae and Allosauroidea. The main areas of phylogenetic uncertainty in the strict consensus tree are found in the interrelationships within megalosaurids and allosauroids. Within the former, all taxa are placed in a polytomy, with the exception of *Streptospondylus*, which is found as the most basal taxon within the clade. In allosauroids, *Xuanhanosaurus* is found as the most basal taxon, followed by piatnitzkysaurids and a polytomy including the majority of other allosauroids, including *Asfaltovenator*. Only metriacanthosaurids and neovenatorids are found as monophyletic groups within this polytomy in allosauroids.

*Reduced consensus*. The IterPCR method was used to identify a number of unstable (mainly fragmentary) basal tetanuran taxa. For the interrelationships of basal allosauroids, *Lourinhanosaurus,* *Saurophaganax,* and *Poekilopleuron* were found to be unstable among the MPTs. *A posteriori* removal of these taxa results in a considerably better resolved Allosauroidea, with *Asfaltovenator* representing the sister taxon to a clade containing Metriacanthosauridae and Allosauria (Fig. 3A). The latter clade is furthermore divided into Allosauridae (with only *Allosaurus* in the reduced consensus tree) and Carcharodontosauria, as found by Carrano et al.^2^ and Rauhut et al^4^.

The problematic *Lourinhanosaurus* was recovered as a coelurosaur by Carrano et al.^2^ and Rauhut et al.^4^, but is consistently found as a basal allosauroid in the current analysis. However, its exact position varies between a basal position outside *Asfaltovenator* and more derived allosauroids and slightly more derived positions as sister taxon to Metriacanthosauridae and Allosauria or as a basal metriacanthosaurid. The poorly known *Saurophaganax* is found as either a basal allosauroid outside *Asfaltovenator* or in several more derived positions, including as sister taxon to Metriacanthosauridae and Allosauria, as sister taxon to Allosauria, as an allosaurid (as in Carrano et al.^2^) or as the basalmost carcharodontosaurian. *Poekilopleuron* was recovered as a megalosaurid by Carrano et al.^2^ and Rauhut et al.^4^, but is consistently found as an allosauroid here. Interestingly, whereas one possible position for this taxon is as sister taxon to Metriacanthosauridae + Allosauria, all other possible positions for this taxon are within Carcharodontosauria, which would make *Poekilopleuron* the possibly oldest representative of this clade.

Support analysis

The Bremer support analysis when all taxa are included show many collapsed nodes and multiple clades with minimal support. Similarly, a jackknife analysis was performed in TNT (100 replicates, summarized as absolute frequencies) and also shows multiple nodes with jackknife values below 50%. Figure S2 shows Bremer (left) and jackknife (right) trees for the analyses including all taxa.

Taxon instability certainly affects measures of support so unstable taxa were also detected among suboptimal trees and trees derived from the jackknife pseudoreplicates. A set of 20 taxa was identified as highly unstable among these trees. We then performed another round of analyses of Jackknife and Bremer Support including all taxa in the searchers but ignored the position of the 20 unstable taxa for summarizing the results of the support measures (i.e., using reduced consensus trees for summarizing results). The 20 taxa detected are unstable are: *Afrovenator, Carcharodontosaurus, Chilantaisaurus, Concavenator, Dubreuillosaurus, Duriavenator, Ichthyovenator, Leshansaurus, Lourinhanosaurus, Magnosaurus, Megaraptor, Metriacanthosaurus, Monolophosaurus, Piveteausaurus, Poekilopleuron, Saurophaganax, Shaochilong, Shidaisaurus, Streptospondylus,* and *Xuanhanosaurus*. Figure S3 shows Bremer (left) and jackknife (right) trees for the analyses using reduced consensus trees that ignore the positions of the above mentioned 20 taxa.

The support analysis ignoring the position of unstable taxa reveal a core of clades that are relatively well supported (i.e., Bremer values >= 3 and jackknife values > 75), such as Carnosauria, a core group of megalosaurids, a core group of spinosaurids, and allosaurians. More specifically, when alternative placements of taxa are evaluated, we find the monophyly of Carnosauria is rather well supported, with the alternative "traditional" Avetheropoda (Coelurosauria + Allosauroidea) requiring at least 9 additional steps (if *Asfaltovenator* and Piatnitzkysauridae are placed outside Avetheropoda), or 10 steps (if *Asfaltovenator* stays inside Allosauroidea). On the other hand, the paraphyly of the "traditional" Megalosauroidea (Megalosauridae + Spinosauridae) is much more poorly supported, with the "traditional" arrangement requiring only two additional steps. Finally, placing Piatnitzksauridae or Piatnitzksauridae + *Asfaltovenator* in a traditional Megalosauroidea requires 4 additional steps.

Analyses excluding *Asfaltovenator*

The strict consensus tree of the analysis excluding *Asfaltovenator* also found a monophyletic Carnosauria, but with limited resolution within this clade (Fig. S4). Monophyletic clades placed in a large basal polyphly with several separate genera within Carnosauria include Spinosauridae + *Monolophosaurus*, Piatnitzkysauridae, Allosauria, and the genus *Yangchuanosaurus*.

Reduced consensus techniques excluding unstable taxa identified by IterPCR retained a monophyletic Megalosauroidea that includes Piatnitzksauridae, Spinosauridae and Megalosauridae, and a monophyletic Allosauroidea, including Metriacanthosauridae and Allosauria (Fig. S4).

Looking at clade support, the "traditional" Avetheropoda now needs only five additional steps, and thus less than a placement of Spinosauridae outside a Megalosauridae + Allosauridae clade (6 additional steps). Placing Piatnitzkysauridae inside Allosauroidea requires only three additional steps. These results show that the inclusion of *Asfaltovenator* has a profound impact on our understanding of basal tetanuran relationships, but also highlights phylogenetic uncertainty at the base of Tetanurae and within Carnosauria.

Synapomorphy list

The following list of unambiguous synapomorphies is given for the nodes present in the reduced consensus tree of the complete analysis (including all taxa). See Fig. S5 for the reduced consensus tree with labeled nodes.

Unambiguous synapomorphies (character numbers follow TNT numeration):

Node 70 :

All trees:

Char. 56: 0 --> 1

Char. 61: 1 --> 2

Some trees:

Char. 11: 12 --> 0

Char. 36: 0 --> 1

Char. 193: 01 --> 2

Char. 296: 0 --> 1

Node 71 :

All trees:

Char. 62: 0 --> 1

Char. 76: 0 --> 1

Char. 123: 1 --> 0

Char. 159: 1 --> 2

Char. 168: 0 --> 1

Char. 187: 0 --> 1

Char. 291: 1 --> 2

Char. 304: 1 --> 2

Some trees:

Char. 34: 1 --> 0

Char. 158: 0 --> 1

Char. 201: 1 --> 2

Char. 324: 2 --> 3

Node 72 :

All trees:

Char. 63: 0 --> 1

Char. 69: 0 --> 1

Char. 93: 0 --> 1

Char. 97: 1 --> 0

Char. 136: 0 --> 1

Char. 138: 0 --> 1

Char. 170: 1 --> 0

Char. 189: 1 --> 0

Char. 195: 0 --> 1

Char. 218: 0 --> 1

Char. 271: 1 --> 2

Char. 272: 0 --> 1

Char. 290: 0 --> 1

Char. 291: 0 --> 1

Char. 326: 1 --> 2

Node 73 :

All trees:

Char. 13: 1 --> 0

Char. 16: 0 --> 1

Char. 25: 0 --> 1

Char. 55: 1 --> 0

Char. 61: 0 --> 1

Char. 116: 1 --> 0

Char. 147: 0 --> 1

Char. 167: 0 --> 1

Char. 183: 0 --> 1

Char. 224: 1 --> 0

Char. 254: 0 --> 1

Char. 311: 0 --> 1

Char. 312: 0 --> 1

Char. 327: 0 --> 2

Some trees:

Char. 28: 0 --> 1

Node 74 :

All trees:

Char. 34: 0 --> 1

Char. 35: 0 --> 1

Char. 38: 0 --> 1

Char. 72: 0 --> 1

Char. 80: 0 --> 2

Char. 85: 0 --> 1

Char. 87: 0 --> 1

Char. 285: 0 --> 1

Char. 318: 0 --> 1

Node 75 :

All trees:

No synapomorphies

Node 76 :

All trees:

Char. 224: 0 --> 1

Char. 226: 0 --> 2

Char. 236: 0 --> 1

Node 77 :

All trees:

Char. 54: 0 --> 1

Char. 55: 0 --> 1

Char. 63: 1 --> 0

Char. 77: 0 --> 1

Char. 128: 1 --> 0

Char. 167: 1 --> 0

Char. 202: 0 --> 1

Node 78 :

All trees:

Char. 91: 0 --> 1

Char. 133: 0 --> 1

Char. 189: 0 --> 1

Char. 264: 0 --> 1

Char. 267: 1 --> 0

Node 79 :

All trees:

Char. 4: 0 --> 1

Char. 11: 12 --> 3

Char. 22: 2 --> 01

Char. 33: 0 --> 1

Char. 49: 2 --> 0

Char. 80: 2 --> 0

Char. 116: 0 --> 1

Char. 127: 0 --> 2

Char. 155: 1 --> 2

Char. 176: 0 --> 1

Node 80 :

All trees:

Char. 303: 0 --> 1

Char. 304: 0 --> 1

Char. 307: 0 --> 1

Node 81 :

All trees:

Char. 269: 0 --> 1

Char. 271: 0 --> 1

Node 82 :

All trees:

Char. 11: 0 --> 1

Char. 35: 1 --> 0

Char. 43: 0 --> 1

Char. 94: 1 --> 0

Char. 95: 1 --> 2

Char. 97: 0 --> 1

Char. 167: 0 --> 1

Char. 221: 0 --> 1

Char. 222: 0 --> 1

Char. 333: 0 --> 1

Char. 338: 0 --> 1

Node 83 :

All trees:

Char. 67: 0 --> 1

Char. 69: 1 --> 0

Char. 319: 0 --> 1

Node 84 :

All trees:

Char. 30: 0 --> 1

Char. 81: 0 --> 2

Char. 84: 0 --> 1

Char. 86: 0 --> 1

Char. 208: 1 --> 0

Char. 239: 1 --> 0

Char. 301: 0 --> 1

Char. 312: 0 --> 1

Char. 332: 0 --> 1

Node 85 :

All trees:

Char. 26: 0 --> 1

Char. 35: 0 --> 1

Char. 41: 0 --> 1

Char. 49: 0 --> 2

Char. 66: 0 --> 1

Char. 125: 0 --> 1

Char. 155: 0 --> 1

Char. 194: 0 --> 1

Char. 201: 0 --> 1

Char. 229: 0 --> 1

Char. 256: 0 --> 1

Char. 302: 0 --> 1

Node 86 :

All trees:

Char. 2: 0 --> 1

Char. 3: 0 --> 1

Char. 22: 1 --> 2

Char. 40: 0 --> 1

Char. 69: 0 --> 1

Char. 74: 0 --> 1

Char. 75: 0 --> 1

Char. 94: 0 --> 1

Char. 95: 0 --> 1

Char. 105: 0 --> 1

Char. 151: 0 --> 1

Char. 177: 0 --> 1

Char. 196: 0 --> 1

Char. 211: 0 --> 1

Char. 212: 1 --> 0

Char. 216: 0 --> 1

Char. 248: 0 --> 1

Char. 249: 0 --> 1

Char. 252: 0 --> 1

Char. 263: 0 --> 1

Char. 264: 1 --> 0

Char. 267: 0 --> 1

Char. 273: 0 --> 1

Char. 274: 0 --> 1

Char. 277: 0 --> 1

Char. 278: 0 --> 1

Char. 298: 0 --> 1

Char. 337: 0 --> 1

Char. 339: 0 --> 1

Char. 340: 0 --> 1

Char. 342: 0 --> 1

Char. 346: 0 --> 1

Char. 347: 0 --> 1

Node 87 :

All trees:

Char. 227: 0 --> 1

Node 88 :

All trees:

Char. 345: 0 --> 1

Node 89 :

All trees:

Char. 272: 1 --> 2

Some trees:

Char. 190: 0 --> 1

Char. 258: 0 --> 1

Char. 259: 0 --> 1

Char. 261: 0 --> 1

Char. 317: 0 --> 1

Char. 323: 0 --> 1

Node 90 :

All trees:

Char. 21: 0 --> 1

Some trees:

Char. 29: 0 --> 1

Node 91 :

All trees:

Char. 83: 0 --> 1

Char. 184: 0 --> 1

Char. 306: 1 --> 0

Node 92 :

All trees:

Char. 314: 0 --> 1

Node 93 :

All trees:

Char. 185: 0 --> 1

Node 94 :

All trees:

Char. 145: 0 --> 1

Some trees:

Char. 141: 0 --> 1

Char. 148: 0 --> 1

Char. 150: 0 --> 1

Char. 151: 1 --> 2

Char. 153: 0 --> 1

Node 95 :

All trees:

Char. 0: 0 --> 1

Char. 1: 1 --> 0

Char. 5: 1 --> 2

Char. 7: 0 --> 1

Char. 10: 0 --> 1

Char. 11: 3 --> 4

Char. 12: 0 --> 1

Char. 14: 0 --> 1

Char. 24: 2 --> 0

Char. 41: 1 --> 0

Char. 47: 0 --> 1

Char. 82: 0 --> 1

Char. 94: 0 --> 2

Char. 98: 01 --> 2

Char. 101: 0 --> 1

Char. 139: 0 --> 1

Char. 142: 0 --> 2

Char. 144: 0 --> 1

Char. 149: 1 --> 3

Char. 178: 0 --> 1

Char. 181: 0 --> 1

Char. 193: 1 --> 2

Node 96 :

All trees:

Char. 336: 0 --> 1

Node 97 :

All trees:

Char. 176: 1 --> 0

Char. 298: 0 --> 1

Node 98 :

All trees:

Char. 182: 0 --> 1

Node 99 :

All trees:

Char. 31: 0 --> 1

Some trees:

Char. 70: 0 --> 1

Char. 84: 1 --> 0

Char. 107: 0 --> 1

Node 100 :

All trees:

Char. 22: 1 --> 0

Some trees:

Char. 80: 2 --> 0

Char. 96: 2 --> 4

Node 101 :

All trees:

Char. 1: 1 --> 2

Char. 8: 0 --> 1

Char. 30: 1 --> 0

Char. 33: 0 --> 2

Char. 53: 0 --> 1

Char. 72: 0 --> 1

Char. 80: 2 --> 1

Char. 90: 0 --> 2

Char. 107: 0 --> 1

Char. 112: 1 --> 0

Char. 126: 0 --> 1

Char. 138: 0 --> 1

Char. 151: 1 --> 0

Char. 157: 0 --> 2

Char. 196: 1 --> 2

Char. 197: 0 --> 1

Char. 198: 0 --> 1

Char. 206: 0 --> 2

Char. 224: 0 --> 1

Char. 260: 0 --> 1

Char. 264: 0 --> 1

Char. 267: 1 --> 0

Char. 293: 0 --> 1

Char. 315: 0 --> 1

Char. 321: 0 --> 1

Char. 329: 02 --> 1

Char. 341: 0 --> 1

Char. 349: 0 --> 1

Node 102 :

All trees:

Char. 5: 1 --> 2

Char. 11: 0 --> 3

Char. 13: 0 --> 1

Char. 23: 0 --> 1

Char. 60: 0 --> 1

Char. 148: 0 --> 1

Char. 152: 1 --> 0

Char. 157: 0 --> 1

Char. 178: 1 --> 2

Char. 207: 0 --> 1

Char. 210: 0 --> 1

Char. 226: 0 --> 2

Char. 276: 0 --> 1

Char. 280: 0 --> 1

Char. 282: 0 --> 1

Char. 295: 0 --> 1

Char. 300: 0 --> 1

Char. 341: 0 --> 1

Node 103 :

All trees:

Char. 22: 2 --> 1

Char. 32: 1 --> 0

Char. 148: 0 --> 1

Char. 155: 1 --> 2

Char. 294: 0 --> 1

Char. 303: 1 --> 2

Char. 309: 0 --> 1

Node 104 :

All trees:

Char. 172: 0 --> 1

Char. 267: 1 --> 0

Char. 326: 1 --> 2

Node 105 :

All trees:

Char. 2: 1 --> 0

Char. 60: 0 --> 1

Char. 66: 1 --> 0

Char. 81: 2 --> 0

Char. 178: 0 --> 1

Char. 270: 0 --> 1

Char. 271: 1 --> 2

Char. 292: 0 --> 1

Char. 318: 0 --> 1

Char. 345: 0 --> 1

Node 106 :

All trees:

Char. 155: 2 --> 1

Char. 178: 0 --> 1

Char. 184: 0 --> 1

Node 107 :

All trees:

Char. 140: 0 --> 1

Char. 189: 1 --> 2

Node 108 :

All trees:

Char. 264: 1 --> 0

Node 109 :

All trees:

Char. 202: 1 --> 0

Char. 281: 1 --> 0

Char. 301: 0 --> 1

Node 110 :

All trees:

Char. 5: 1 --> 0

Char. 11: 1 --> 0

Char. 27: 0 --> 1

Char. 56: 0 --> 1

Char. 73: 1 --> 2

Char. 114: 1 --> 0

Char. 137: 1 --> 0

Char. 162: 0 --> 1

Char. 164: 1 --> 0

Char. 248: 3 --> 2

Char. 263: 1 --> 0

Char. 300: 0 --> 1

Some trees:

Char. 46: 0 --> 1

Node 111 :

All trees:

Char. 178: 0 --> 2

Char. 194: 1 --> 0

Char. 206: 2 --> 1

Char. 225: 0 --> 1

Char. 280: 0 --> 1

Char. 345: 0 --> 1

Node 112 :

All trees:

Char. 228: 0 --> 1

Char. 237: 0 --> 1

Char. 276: 0 --> 1

Char. 331: 0 --> 1

Char. 334: 0 --> 1

Node 113 :

All trees:

Char. 40: 1 --> 2

Char. 54: 0 --> 1

Char. 60: 0 --> 1

Char. 65: 0 --> 1

Char. 171: 1 --> 2

Char. 205: 0 --> 1

Char. 350: 0 --> 1

Node 114 :

All trees:

Char. 1: 1 --> 2

Char. 5: 1 --> 0

Char. 11: 2 --> 3

Char. 38: 0 --> 3

Char. 122: 0 --> 1

Node 115 :

All trees:

Char. 15: 0 --> 1

Char. 18: 0 --> 1

Char. 116: 1 --> 0

Char. 203: 0 --> 1

Node 116 :

All trees:

Char. 193: 1 --> 2

Node 117 :

All trees:

Char. 262: 0 --> 2

Node 118 :

All trees:

Char. 287: 0 --> 1

Char. 295: 0 --> 1

Results

All characters are taken from Rauhut et al.^4^ if not otherwise indicated. For presacral vertebrae, we used the slightly modified character definitions and codings from Evers et al^56^. Several logically ordered characters were treated as such.

1. Premaxilla, inter-premaxillary suture in adults: open (0), fused (1).
2. Premaxilla, height/length ratio ventral to external naris (length measured along ventral border of bone): < 0.5 (0), 0.5-0.99 (1), 1-1.49 (2), 1.5 or higher (3) ordered
3. Premaxilla, subnarial process and ventral border of naris: contacts nasals, excluding maxilla from narial margin (0), reduced and separate from nasals by maxillary contribution to narial margin (1).
4. Premaxilla, posterior extent of nasal process relative to posterior tip of subnarial process: considerably more anterior (0); even (1); posterior (2). ordered
5. Premaxilla, form of premaxilla-nasal suture: V-shaped (0), W-shaped (1).
6. Position of anterior end of external nares: over anterior half of ventral margin of premaxillary body (0); over the posterior half of the ventral margin of the premaxillary body (1); posterior to the ventral margin of the premaxillary body (2). ordered
7. Premaxilla, diastema (‘subnarial gap’) adjacent to maxilla along dentigerous margin: absent (0), present (1).
8. Premaxilla, mediolateral constriction of posterior portion: absent (0), present (1).
9. Premaxilla, development of subnarial (maxillary) process: well-developed, rod or plate-like (0), reduced to a short, stout triangle, as long as or shorter than its basal width (1).
10. Subnarial foramen on the premaxilla–maxilla suture: absent (0), present (1).
11. Premaxilla, articulation with maxilla: planar (0), interlocking (1).
12. Anterior ramus of the maxilla: absent, anterior margin of maxillary body confluent with anterior margin of the ascending process (0); present but very short (length/height ratio less than 1) (1); present moderately long (1-1.35) (2); present, long (1.35-1.7) (3); present, very long (more than 2) (4). ordered
13. Maxilla, orientation of anteriormost alveolus: vertical (0), angled anteriorly (1).
14. Anterodorsal margin of the ascending process of the maxilla: straight or gently curved (0), with pronounced kink at about mid-length, with a more anteriorly facing margin ventral and a more dorsally facing margin dorsal to the kink (1).
15. Maxilla, morphology of palatal process: long, ridged or fluted prong (0), long and plate-shaped (1).
16. Maxilla, position of palatal process: ventral, immediately dorsal to paradental plates (0), dorsal, immediately ventral to dorsal surface of maxillary anterior ramus (1).
17. Maxilla, horizontal ridge (prominent ‘lingual bar’) between palatal process and antorbital fenestra: absent (0), present (1).
18. Maxilla, depth of paradental plates relative to anteroposterior width: low, < 1.8 (0); tall > 1.8 (1).
19. Maxilla, ventral extent of paradental plates relative to lateral wall: as far ventral (0); fall short (1).
20. Maxilla, arrangement of nutrient foramina on lateral surface: single row or no distinct pattern (0); a second, more dorsally placed row is present anteriorly and converges with the ventral row posteriorly (1); a second, dorsally placed row is present and extends posteriorly more or less parallel to the ventral row (2).
21. Maxilla, anteroventral border of antorbital fossa: graded or stepped (0), demarcated by raised ridge (1).
22. Maxilla, anterior margin of antorbital fossa: rounded (0); squared (1).
23. Maxilla, ventral extent of antorbital fossa (as measured from the rim of the antorbital fenestra to the highest point of the ridge marking its border) at the level of about the half length of the antorbital fenestra: small or absent, less than 1/3 the height of the maxillary body at this level (0); moderate, between 1/3 and half the height (1), dorsoventrally deep, more than half the height (2). ordered
24. Maxilla, position of anterior end of antorbital fossa: posterior to or level with posterior rim of external nares (0), ventral to external nares (1).
25. Medial wall of the anterior end of the maxillary antorbital fossa: lacking depressions or foramina (0); with a large depression without sharply defined margins (1); with a sharply rimmed maxillary fenestra (2).
26. Development of maxillary fenestra: opens medially into a small maxillary antrum with a robust medial wall (0); opens medially into a large maxillary antrum that is medially open or only covered by a very thin bony wall (1); opens anteriorly into a large antrum within the ascending process of the maxilla (2) This character is inapplicable in taxa that lack a maxillary fenestra.
27. Promaxillary foramen: absent (0), present and opens anteriorly into pneumatic recesses in the ascending process of the maxilla (1).
28. Size of the promaxillary foramen in relation to the maxillary fenestra: smaller (0); larger (1). This character is inapplicable in taxa that lack a maxillary fenestra.
29. Maxilla, development of pneumatic fossa (excavatio pneumatica) in ascending process: absent (0), present (1).
30. Maxilla, pneumatic region on medial side of maxilla posteroventral to maxillary fenestra: absent (0); present (1).
31. Maxilla, posterior end of tooth row relative to orbit: beneath (0), anterior (1).
32. Maxilla and nasal, external surface texture: smooth (0), sculptured (1).
33. Nasal, inter-nasal contact in adults: separate (0), partly or fully fused (1).
34. Nasal, posterior narial margin: absent or weak fossa (0), large fossa (1), laterally splayed hood (2).
35. Posteriorly pointed, sharply rimmed depression on the lateral side of the nasal posterodorsal to the external nares: absent (0), present (1).
36. Nasal, participation in antorbital fossa: absent or at edge (0), present (1).
37. Nasal, antorbital fossa in lateral view: visible (0); occluded by ventrolaterally overhanging lamina (1).
38. Nasal, pneumatic foramina: absent (0), present (1).
39. Nasal, development of dorsolateral surfaces: none, nasals low and dorsally convex (0), pronounced dorsolateral rims, sometimes with lateral crests (1), tall, parasagittal crests (2), inflated and forming a hollow midline crest (3).
40. Nasal, sculpturing: low rugosity (0), deeply rugose, bears large excresences (1) [inapplicable in taxa that lack craniofacial rugosity].
41. Antorbital fossa and dorsal rim on the anterior process of the lacrimal: present, but fossa is largely hidden in lateral view by an overhanging lateral lamina and only exposed anteriorly (0); present, widely exposed laterally and confluent with the antorbital fossa of the dorsal part of the ventral process (1); dorsal rim absent, no differentiation between fossa and rim on the lateral surface of anterior process (2).
42. Lacrimal, morphology of lateral lamina of ventral process of lacrimal: forming a continuous sheet of bone between the ventral and anterior processes (0); invaginated dorsally and convex anteriorly, anteriormost point situated dorsal to midheight of ventral process (1); anteriormost point situated around midheight of ventral process (2).
43. Lacrimal, dorsal and ventral portions of antorbital fossa: separated by anterior projection of lateral lamina (0), continuous, lateral lamina does not project far anteriorly (1).
44. Lacrimal fenestra: absent (0); present (1).
45. Lacrimal, openings in lacrimal recess: single (0), multiple (1).
46. Lacrimal horn: absent (0); small dorsal rugosity (1); low, broad, rugose bar (2); large triangular horn (3). ordered
47. Lacrimal, suborbital process: absent (0), present (1).
48. Lacrimal, angle between anterior and ventral rami: ~90° (0), < 75° (1).
49. Lacrimal, length of anterior process relative to ventral process: more (0), or less than 80% (1).
50. Jugal, position of anterior end: posterior to internal antorbital fenestra, but reaching its posterior rim (0), excluded from internal antorbital fenestra (1), expressed at rim of internal antorbital fenestra, with distinct anterior process extending beneath it (2).
51. Jugal, pneumatisation: absent (0), internally hollowed and transversely inflated by foramen in posterior rim of antorbital fossa (1).
52. Jugal, antorbital fossa: absent (0), present (1).
53. Anterior end of jugal: slender and not or only slightly expanded (0); strongly expanded, and expansion forms at least a small part of the anterior margin of the orbit (1).
54. Jugal, orientation of orbital margin: angled posterodorsally (0), vertical (1).
55. Dorsoventral height of the posterior process of jugal: less than (0) or subequal or more than dorsoventral height of suborbital part (1).
56. Postorbital, articulation with jugal: planar or with a shallow, V-shaped groove dorsally (0), with a deep, broad groove posteriorly, ventral process with U-shaped cross-section (1).
57. Postorbital, suborbital flange: absent (0), present (1).
58. Development of suborbital flange of postorbital: small, triangular eminence (0), dorsoventrally elongate, large rounded flange (1), jugal process curved anteroventrally and suborbital process developed as large, sharply angled, triangular flange (2) This character is not applicable to taxa that do not have a suborbital flange.
59. Postorbital, ventral extent relative to ventral margin of orbit: substantially above (0), approximately same level (1).
60. Medial side of posterior process of the postorbital: straight or concave, dorsal margin forms a sharp rim (0); convex, medial margin curves into dorsal margin (supratemporal fossa extends onto process) (1).
61. Supratemporal fossa on the anterior process of the postorbital: forms a large shelf on the dorsal surface of the process (0); reduced, restricted to the posteriormost part of the anterior process and faces more posterodorsally than dorsally (1).
62. Supraorbital brow: absent, anterior end of postorbital tapers (0); present as a dorsoventrally expanded, anteriorly rounded, rugose swelling over the posterior part of the orbit (1); large, strongly rugose supraorbital brow (possible formed by a separate palprebal ossification fused to the postorbital) that connects the postorbital with the lacrimal present (2).
63. Posterior process of the postorbital: Pointed and transversely narrow (0); broadened transversely posteriorly and wider than high (1).
64. Parietal-postorbital contact at the anterior end of the supratemporal fenestra: absent, parietal and postorbital separated by posterior process of frontal that reaches the laterosphenoid (0); present, frontal excluded from laterosphenoid (1).
65. Prefrontal in adult individuals: Exposed on the anterodorsal margin of the orbit (0), reduced, not exposed at the anterior margin of the orbit, might only be visible at the dorsal margin of the orbit (1), absent as separate ossification (2). (modified from Carrano et al.^2^ and Rauhut et al.^4^)
66. Prefrontal, articulation with frontal: planar (0), peg-and-socket (1).
67. Frontal, exposure along orbital rim: broad, one third or more of the dorsal orbital margin (0), reduced, less than one third of the dorsal orbital margin (1). (modified from Carrano et al.^2^ and Rauhut et al.^4^)
68. Parietal, articulation with supraoccipital: abuts (0), overlaps (1).
69. Parietal, development of median skull table between supratemporal fossae: flat, but relatively narrow (less than 50% of width of supratemporal fenestrae) (0), separated by a triangular plate of bone anteriorly, but narrowing to a sagittal crest posteriorly (1), narrow with sharp sagittal crest (2), very broad, widely separating upper temporal fenestrae, skull table at least 50% of width of supratemporal fenestra and fossa (3). (modified from Carrano et al.^2^ and Rauhut et al.^4^)
70. Parietal, size and elevation of nuchal wedge and alae: small to moderate, height of alae less than height of base of paroccipital process (0), expanded, height more than height of base of paroccipital process (1). (modified from Carrano et al.^2^ and Rauhut et al.^4^)
71. Supratemporal fossa, anteromedial corner: open dorsally (0); partially roofed over by a small shelf of the frontalparietal (1).
72. Squamosal, constriction of lower temporal fenestra: absent (0), present (1).
73. Squamosal, anterodorsal lamina: emarginated by upper temporal fenestra (0); continuous (1).
74. Squamosal, flange covering quadrate head laterally: absent (0), present, covers the posterior part of the head and separated from quadratojugal process of squamosal by a wide, U-shaped incision (1), present, covers most of quadrate head and separated from quadratojugal process only by a narrow, slit-like incision (2). ordered (modified from Carrano et al.^2^ and Rauhut et al.^4^)
75. Squamosal, articulation with quadratojugal: at tip (0), absent (1), broad (2).
76. Quadratojugal, anteriormost point of ventral process relative to lower temporal fenestra: ventral, no further than two thirds of the length of the fenestra from the posterior border (0), further anterior, subequal to the anterior border of the fenestra (1). (modified from Carrano et al.^2^ and Rauhut et al.^4^)
77. Quadrate, pneumatisation: absent (0), present (1).
78. Position of the quadrate head in relation to the orbit: low, below two-thirds of the height of the orbit (0), at two-thirds of the height of the orbit or higher (1) (modified from Carrano et al.^2^ and Rauhut et al.^4^)
79. Angle between quadrate ridge (sensu Hendrickx et al.^57^) and distal quadrate condyles: more than 70° (0), less than 70° (1). (modified from Carrano et al.^2^ and Rauhut et al.^4^)
80. Proportion of the length of the quadrate that is occupied by the pterygoid wing: less than 70% (0) or 70% or more (1). (modified from Carrano et al.^2^ and Rauhut et al.^4^)
81. Quadrate foramen: developed as a distinct opening between the quadrate and quadratojugal (0); absent(1); mainly or entirely enclosed in the quadrate (2). (modified from Carrano et al.^2^ and Rauhut et al.^4^)
82. Orientation of the quadrate in lateral view: more or less vertical, mandibular joint approximately straight below quadrate head (0), anteroventrally inclined, mandibular joint notably anterior to quadrate head (1), posteroventrally inclined, mandibular joint notably posterior to quadrate head (2). (modified from Carrano et al.^2^ and Rauhut et al.^4^)
83. Quadrate, head shape in dorsal view: oval (0), subrectangular (1).
84. Quadrate, medial foramina adjacent to condyles: absent (0), present (1).
85. Ventral rim of the basis of the paroccipital processes: above or level with the dorsal border of the occipital condyle (0); situated at mid-height of occipital condyle or lower (1). (modified from Carrano et al.^2^ and Rauhut et al.^4^)
86. Paroccipital processes: directed laterally or slightly ventrolaterally (0); directed strongly ventrolaterally, with distal end entirely below the level of the foramen magnum (1). (modified from Carrano et al.^2^ and Rauhut et al.^4^)
87. Supraoccipital, anteroposterior depth of median ridge relative to occipital condyle length: less (0), greater (1).
88. Maximal width of dorsal expansion of supraoccipital ridge: less than or subequal to width of foramen magnum (0), notably greater than width of foramen magnum (1). (modified from Carrano et al.^2^ and Rauhut et al.^4^)
89. Supraoccipital, participation in foramen magnum: present, ventral margin of supraoccipital forms a more or less straight line above the foramen, forming most of its dorsal rim (0), narrow, formed by a small median ventral process of the supraoccipital separating the exoccipitals on the dorsal edge of foramen (1), absent, exoccipitals meet in the midline above the foramen magnum (2). (modified from Carrano et al.^2^ and Rauhut et al.^4^)
90. Basioccipital, ventrolateral pair of pneumatic cavities invading neck of occipital condyle and joining medially: absent (0), present (1).
91. Morphology of posterior basioccipital surface below the condyle: with undivided longitudinal median groove (0), median groove divided dorsally by small median lamina (1), with large longitudinal ridge separating two large lateral depressions (2). (modified from Carrano et al.^2^ and Rauhut et al.^4^)
92. Basioccipital, fossa ventral to occipital condyle in basioccipital apron: narrow and with slightly diverging margins, not exceeding 60 % of the width of the occipital condyle (0), broad with considerably diverging margins, reaching 70% or more of the width of the condyle (1), narrow (less than 60% of condyle width) and with parallel margins (2). (modified from Carrano et al.^2^ and Rauhut et al.^4^)
93. Notch separating a medial basioccipital portion of the basal tubera from a lateral basisohenoid portion: absent (0), present (1). (modified from Carrano et al.^2^ and Rauhut et al.^4^)
94. Width of basioccipital ventral to the occipital condyle: greater than width of occipital condyle (0), subequal to or less than the width of the occipital condyle (1).
95. Basisphenoid, location of basipterygoid processes relative to basal tubera: anterior or slightly anteroventral, basisphenoid recess opens ventrally (0), anteroventrally, basisphenoid recess opens posteroventrally (1), almost directly ventral, basisphenoid recess anteroposteriorly narrower than wide and opens more posteriorly than ventrally (2).
96. Basisphenoid, presence and depth of basisphenoid recess: absent (0), shallow, longer than deep (1), deep, deeper than long (2). ordered (modified from Carrano et al.^2^ and Rauhut et al.^4^)
97. Basisphenoid, shape of opening for basisphenoid recess: elongate oval (0), teardrop-shaped, narrowing posteriorly (1), subcircular (2), anteroposteriorly compressed, slit-like (3), trapezoidal, widening posteriorly (4). (modified from Carrano et al.^2^ and Rauhut et al.^4^)
98. Posterior part of the ventral margin of the basisphenoid between the basal tubera and the basipterygoid processes: gently concave in lateral view (0), straight or slightly convex in lateral view (1). (modified from Carrano et al.^2^ and Rauhut et al.^4^)
99. Basipterygoid processes: on elongate stalks, with rather small articular surface (0), broad, enlarged articular surface facing anteroventrolaterally at lateral sides of the anterior end of the basisphenoid (1), broad, on stout stalks, strictly ventrolaterally directed (2). (modified from Carrano et al.^2^ and Rauhut et al.^4^)
100. Exit of cranial nerves X and XI: laterally through the metotic foramen (0), posteriorly through a foramen in the paracondylar recess (1). (modified from Carrano et al.^2^ and Rauhut et al.^4^)
101. Subcondylar recess on the occiput: small, restricted to the occiput lateral to the occipital condyle (0), extended ventrally, developed as deep depression on the lateral sides of the ventral part of the occiput (1). (modified from Carrano et al.^2^ and Rauhut et al.^4^)
102. Posterior end of dorsal skull table: faces dorsally, more or less straight above the orbit in lateral view (0), parietal faces posterodorsally, frontal vaulted above the orbit (1). (new character)
103. Angle between the posterior end of the dorsal skull roof and the main body of the supraoccipital: approximately 90° (0), notably obtuse (1). (modified from Carrano et al.^2^ and Rauhut et al.^4^)
104. Braincase, morphology of trigeminal foramen: single (0), partly split (1), fully split (2).
105. Exit of the abducens nerve (cranial nerve VI): placed within the pituitary fossa or the depression surrounding it (0), placed lateral to the pituitary fossa and its surrounding depression (1). (modified from Carrano et al.^2^ and Rauhut et al.^4^)
106. Well-developed anterior tympanic recess in the braincase: absent (0), present (1). (modified from Carrano et al.^2^ and Rauhut et al.^4^)
107. Pneumatic recess associated with entrance of the carotid artery on the lateral side of the parabasisphenoid: absent (0), present (1). (modified from Carrano et al.^2^ and Rauhut et al.^4^)
108. Braincase, ossification of interorbital region: weak or absent (0), extensive, ossified sphenethmoid and interorbital septum (1).
109. Length of the anterior, maxillary process of the palatine (as measured from the anterior end of the junction with the vomerine process to the anterior tip) in relation to length of jugal process (as measured from the posterior end of the junction with the pterygoid process to the posterio tip): less or subequal (0), longer (1). (modified from Carrano et al.^2^ and Rauhut et al.^4^)
110. Palatine, pneumatic recess: absent (0), present (1).
111. Pterygoid, pocket on ectopterygoid flange: absent (0), present (1).
112. Anteroventral expansion of jugal process of ectopterygoid: absent (0), present (1) (new). Allain^14^ proposed the presence of an anteroventral (or ventral) process of the jugal process of the ectopterygoid as an autapomorphy of *Dubreuillosaurus valesdunensis*. However, an anteroventral expansion, resulting in a small process is also present in *Allosaurus* (Saurier Museum Aathal, SMA 0006), and *Asfaltovenator* shows an anteroventral process that is quite similar to that found in *Dubreuillosaurus*.
113. Ectopterygoid, ventral fossa: absent (0), present (1).
114. Ventral fossa of the ectopterygoid: simple depression (0), invaginates the lateral ectopterygoid body (1). (modified from Carrano et al.^2^ and Rauhut et al.^4^)
115. Size of external mandibular fenestra: Large, surangular above the fenestra accounts for less than half of the height of the mandible (0), reduced, surangular accounts for more than half the height of the mandible (1). (modified from Carrano et al.^2^ and Rauhut et al.^4^)
116. Mandible, position of anterior end of external mandibular fenestra relative to last dentary tooth: posterior (0), ventral (1).
117. Dorsoventral expansion at anterior end of the dentary tooth row: absent (0), present (1). (modified from Carrano et al.^2^ and Rauhut et al.^4^)
118. Shape of the anterior end of the dentary: rounded (dorsoventrally convex) (0), squared (dorsoventrally straight) (1). (modified from Carrano et al.^2^ and Rauhut et al.^4^)
119. Anteroventral flange or process at the anterior end of the dentary: absent (0), present (1). (modified from Carrano et al.^2^ and Rauhut et al.^4^)
120. Enlarged tooth or teeth in the anterior end of the dentary: absent (0), present, usually in the third and/or fourth dentary alevolus (1). (modified from Carrano et al.^2^ and Rauhut et al.^4^)
121. Dentary, shape in dorsal view: straight (0), curves anteromedially (1).
122. Dentary, paradental groove: narrow along entire length (0), wide anteriorly defining a distinct gap between medial dentary wall and paradental plates (1).
123. Dentary, longitudinal groove housing dorsally situated row of neurovascular foramina on lateral surface: absent or weak (0), present and well-defined (1).
124. Dentary, number of Meckelian foramina: one (0), two (1).
125. Posterior end of the dentary: with dorsal and ventral processes subequal in length or dorsal process slightly longer (0), sloping psteroventrally, ventral end extends considerably further posteriorly than dorsal end (1). (modified from Carrano et al.^2^ and Rauhut et al.^4^)
126. Morphology of posterior margin of dentary: forked (0), posteroventrally sloping margin with incision for mandibular fenestra (1). (modified from Carrano et al.^2^ and Rauhut et al.^4^)
127. Dentary, morphology of surangular articulation just above external mandibular fenestra: small notch (0), large socket (1).
128. Splenial, contour of posterior edge: straight (0), curved (1), notched (2).
129. Splenial, size of splenial (‘mylohyoid’) foramen: small (height less than 15 % of height of splenial at the level of the foramen) (0), intermediate (height between 15 % and 25 % (1), large (height 25% or more) (2). ordered (modified from Carrano et al.^2^ and Rauhut et al.^4^)
130. Splenial, foramen in ventral part: completely enclosed by bone (0), open anteroventrally (1).
131. Surangular, horizontal ridge on lateral surface below mandibular joint: weak or absent (0), strong (1).
132. Surangular, number of posterior surangular foramina: one (0), two (1).
133. Angular ventral projection below the foramen for the chorda tympani on the medial side of the articular at the posteriomedial margin of the mandibular glenoid: absent, ventromedial margin of glenoid rounded (0), present (1). (modified from Carrano et al.^2^ and Rauhut et al.^4^)
134. Anterior rim of the mandibular glenoid in lateral view: confluent with the dorsal margin of the surangular (0), raised above the dorsal margin of the surangular (1). (modified from Carrano et al.^2^ and Rauhut et al.^4^)
135. Ossified antarticular in the mandible: absent (0), present (1) (new character^11^)
136. Retroarticular process of the mandible: elongate, as long as or longer than anteroposterior length of mandibular glenoid (0), short, shorter than length of mandibular glenoid (1). (modified from Carrano et al.^2^ and Rauhut et al.^4^)
137. Retroarticular process, mediolateral width relative to posterior width of dentary: ≤ (0), > (1).
138. Retroarticular process, orientation of attachment surface: posterodorsal (0), posterior (1).
139. Paradental plates, continuity and replacement groove: separated, groove present (0), forming a continuous medial lamina (‘fused’), groove absent (1).
140. Interdental plates, visibility in medial view: widely exposed, subpentagonal and moderate–tall (0), obscured by an expanded paradental lamina, triangular apices only may be visible (1).
141. Paradental plates, surface texture: smooth (0), vertically striated or ridged (1).
142. Lateral maxillary and dentary teeth: recurved, so that tip of the tooth is placed distal to distal carina, distal carina concave (0); straight or almost straight, tip of tooth placed mesial to distal carina, distal carina straight or convex (1). (modified from Carrano et al.^2^ and Rauhut et al.^4^)
143. Teeth, crown striations: absent (0), present on premaxillary and/or anterior dentary teeth only (1), present on all tooth crowns (2). (modified from Carrano et al.^2^ and Rauhut et al.^4^)
144. Teeth, enamel wrinkles: absent (0), pronounced marginal enamel wrinkles (1). (modified from Carrano et al.^2^ and Rauhut et al.^4^)
145. Teeth, mid-crown cross-section: elliptical (0), circular (1).
146. Teeth, maxillary and dentary, serrations: present (0), absent (1).
147. Teeth, maxillary and dentary, extent of anterior carina: to base of crown (0), at mid-height of crown or more apically (1).
148. Premaxillary teeth, arrangement of carinae: nearly symmetrical, on opposite sides (0), more asymmetrical, both on lingual side (1).
149. Premaxillary teeth, serrations: present (0), absent (1).
150. Premaxillary teeth, number: three (0), four (1), five (2), more than five (3). ordered (modified from Carrano et al.^2^ and Rauhut et al.^4^)
151. Premaxillary teeth, spacing: even (0), paired and spaced (1).
152. Size of first premaxillary tooth (or alveoli): subequal to second premaxillary tooth (0), less than two thirds of the size of the second premaxillary tooth (1), less than half the size of the second premaxillary tooth (2). ordered (modified from Carrano et al.^2^ and Rauhut et al.^4^)
153. Maxillary teeth, number: > 17 (0), 11–17 (1), < 11 (2). ordered
154. Maxillary teeth, mid-tooth spacing: adjacent (0), with diastemata (1).
155. Dentary teeth, size and number relative to maxillary teeth: approximately equal (0), smaller and approximately 1.5 times as numerous (1).
156. Cervical vertebrae, anterior articular facet: concave (0), flat (1), convex (2). ordered
157. Pneumatic feature posterior or posterodorsa to parapophysis (anterior pleurocoel) in cervical vertebrae: absent (0), large, blind depressions (1), large foramina (2).
158. Pneumatic feature on the posterior half of the vertebral centrum in cervical vertebrae: absent (0), large depression (1), foramen (2).
159. Presacral vertebrae, extent of anterior pleurocoel: anterior dorsals only (0), to sacrum (1).
160. Vertebrae, internal structure of pneumatic centra: absent (0), camerate (1), camellate (2).
161. Epipophysis of the atlantal neural arch in lateral view: slender, rod-like (0), expanded, triangular (1).
162. Axial neural spine: anteroposteriorly extensive, sheet-like, with convex or only gently ascending dorsal margin (0), anteroposteriorly reduced, rod-like, with steeply ascending dorsal margin (1).
163. Axis, orientation of intercentrum ventral surface: horizontal or slightly anteroventral (0), tilted anterodorsally (1).
164. Length of epipophyses of the axis: short, approximately level with posterior end of postzygapophyses (0), long, overhang postzygapophyses posteriorly for more than half the length of the postzygapophyseal articular facet (1).
165. Spinopostzygapophyseal laminae of the axis: Extensive, connecting the spine with the postzygapophyses in a large arch, resulting in a large, triangular to rhomboid fossa on the posterior side of the neural arch (0), reduced, strongly invaginated between spine and postzygapophyses, so that at least the distal part of the spine has parallel borders and the posterior fossa is reduced (1).
166. Development of parapophysis on axis: well-developed facet on the anteroventral side (0), indistinct, or only developed as a slightly roughened patch (1)
167. Development of diapophysis on axis: indistinct, probably absent (0), developed as distinct ventrolateral projection (1).
168. Axis, pleurocoels: absent (0), present (1).
169. Cervical vertebrae, morphology of anterior pleurocoel: single opening (0), two openings oriented anteroventralposterodorsal or very plastic morphology (1).
170. Cervical vertebrae, middle, shape of anterior pleurocoel: round (0), anteroposteriorly elongate (1).
171. Cervical vertebrae, anterior, ventral keel: present (0), absent or weak ridge (1).
172. Cervical vertebrae, anterior, demarcation of dorsal surface of neural arch from diapophyseal surface: gently sloping (0), pronounced edge or ridge that offsets the dorsal from the lateral surface (1), elevated prezygoepipophyseal lamina, dorsal surface concave between neural spine and the lamina (2). ordered
173. Cervical vertebrae, articular surface of prezygapophyses: planar (0), flexed (1).
174. Cervical vertebrae, perimeter of anterior articular surface: not rimmed by a flattened peripheral band (0), flat, forming a distinct rim (1).
175. Cervical vertebrae, anterior, transverse distance between prezygapophyses relative to width of neural canal: < (0), >, prezygapophyses situated lateral to neural canal (1).
176. Cervical vertebrae, anterior, morphology of epipophyses: low, wider than high, posteriorly pointed (0), transversely narrow, high (1), high, robust (2).
177. Neural spine of mid-cervical vertebrae: anteroposteriorly longer than dorsoventrally high (0), higher than long (1).
178. Cervical vertebrae, longest post-axial elements: first five (0), last five (1).
179. Length/posterior height ratio of mid-cervical centra: less than 1.75 (0), 1.75-2.75 (1), more than 2.75 (2). ordered
180. Height of anterior dorsal neural spines (as measured from the dorsal margin of the postzygapophysis): less (0) or more (1) than 1.25 times the height of the neural arch (as measured from the dorsal rim of the centrum to the dorsal margin of the postzygapophysis).
181. Ventral keel in posterior-most cervicals and anterior-most dorsals: ventrally concave, a rounded hypapophysis might be present anteriorly (0), forming a straight to slightly convex ventral margin, anterior end of keel protrudes ventrally from the anterior articular surface and is separated from the latter by a distinct step (1).
182. Dorsal vertebrae, pneumaticity/webbing at base of neural spines in middle to posterior dorsals: absent (0), present (1).
183. Middle to posterior dorsal vertebrae, accessory centrodiapophyseal lamina: absent (0), present (1).
184. Dorsal vertebrae, size of infraprezygapophyseal fossa: small (0), expanded (1).
185. Dorsal vertebrae, anterior, ventral keel: absent or developed as a weak ridge (0), pronounced, around 1/3 the height of centrum and inset from lateral surfaces (1).
186. Dorsal vertebrae, anterior, size of pneumatic foramen in centrum: small (0); enlarged (1).
187. Dorsal vertebrae, elevation of parapophyses: slightly elevated from centrum (0), project far laterally, more than half the diapophyseal length (1).
188. Dorsal vertebrae, orientation of hyposphene laminae: diverge ventrolaterally (0), parallel and sheet-like (1).
189. Dorsal vertebrae, position of parapophyses in posteriormost elements: on the same level as transverse process (0); distinctly below transverse process (1).
190. Dorsal vertebrae, distinct step-like ridge lateral to hyposphene, running posterodorsally from dorsal border of neural canal to posterior edge of postzygapophyses: absent (0); present (1); ridge present and is developed into a prominent lamina that bisects the infrapostzygapophyseal fossa in posterior dorsal vertebrae (2). ordered
191. Dorsal vertebrae, middle and posterior, postzygapophyses with tab-like lateral extensions of articular facets: absent (0); present (1).
192. Dorsal vertebrae, morphology of neural spines: transversely compressed sheets (0), transversely broad anteriorly and posteriorly, central regions of lateral surface embayed by deep vertical troughs (1).
193. Dorsal vertebrae, posterior, inclination of neural spines: vertical or posterior (0), anterior (1).
194. Dorsal vertebrae, height of neural spines relative to centrum height: low, ≤ 1.3x (0), moderate, 1.4-1.8x (1); tall, ≥ 2.0x (2). ordered
195. Middle to posterior dorsal vertebrae, centrum length relative to height: more than 2 (0), less than 2 (1).
196. Sacral vertebrae, centrum pneumaticity: absent (0), pleurocoelous fossae (1); pneumatic foramina (2).
197. Sacral vertebrae, number: 2 [primordial sacrals only] (0), 5 [1 dorsosacral, 2 caudosacrals] (1), 6 [2 dorsosacrals, 2 caudosacrals] (2). ordered
198. Sacral vertebrae, transverse dimensions of middle centra relative to other sacrals: equivalent (0), constricted (1).
199. Sacral vertebrae, orientation of ventral margin of middle centra: approximately horizontal (0), strongly arched (1).
200. Sacral vertebrae, dorsal edge of neural spines: as thin as remainder of spine (0), transversely thickened (1).
201. Sacral vertebrae, pneumaticity of neural arches: weak or absent (0), paired fossa ventral to diapophyses (1).
202. Caudal vertebrae, anterior, morphology of ventral surface: flat (0), groove (1), ridge (2).
203. Caudal vertebrae, L-shaped neural spines: absent (0), present (1).
204. Anterior to mid-caudal vertebrae, depressions or pneumatic foramina in centrum: absent (0), large, pronounced pleurocentral depressions on the dorsal part of the lateral side (1), pneumatic foramina (2).
205. Caudal vertebrae, anterior, centrodiapophyseal laminae on neural arch: weak or lacking (0), as prominent as in dorsal vertebrae, defining deep infradiapophyseal fossa that penetrates neural arch (pneumatic) (1).
206. Caudal vertebrae, anterior, proportions of neural arch base relative to centrum proportions: < (0), ≥ (1).
207. Caudal vertebrae, middle, morphology of neural spines: rod-like and posteriorly inclined (0), subrectangular and sheet-like (1), rod-like and vertical (2).
208. Cervical ribs, articulation with cervical vertebrae in adults: separate (0), fused (1).
209. Cervical ribs, length of anterior process: short (0), long (1).
210. Gastralia, posteriormost gastral segments: separate (0), united into single, boomerang-shaped elements (1).
211. Sacral ribs, articulations in adults: separate (0), fused together (1).
212. Sacral ribs, position of posterior attachment to ilium: ventral (0), posterodorsal (1).
213. Sacral ribs, depth relative to ilium height: < 85% (0), ≥ 90% (1).
214. Chevrons, morphology in middle caudal vertebrae: rodlike or only slightly expanded ventrally (0), L-shaped (1).
215. Chevrons, proximal articular surface: divided into anterior and posterior facets by distinct transverse ridge (0), no ridge, but low lateral mounds may be present, one on each side (1).
216. Chevrons, curvature: straight or gently curved (0), strongly curved (1).
217. Chevrons, anterior process: absent (0); present (1).
218. Chevrons, morphology of distal end in anterior and middle elements: expanded anteroposteriorly (0), unexpanded, tapers ventrally (1).
219. Scapula, angle between blade and acromion: gradual, oblique (0), abrupt, perpendicular (1).
220. Scapula, size of acromion process: moderate (0), marked (1).
221. Scapula, midshaft expansion of blade: absent (0), present (1).
222. Scapula, distal expansion of blade: marked (0), weak/absent (1).
223. Scapula, length:width ratio of blade: ≤ 7 (0), 7.5–9 (1), > 10 (2). ordered
224. Scapulocoracoid, shape of anterior margin: indented or notched between acromial process and coracoid suture (0), smoothly curved and uninterrupted across scapula–coracoid contact (1).
225. Scapulocoracoid, glenoid lip: moderate (0), marked (1).
226. Coracoid, development of posteroventral process: low, rounded posteroventral eminence (0), pronounced, posteroventrally tapering process (1).
227. Coracoid, development of biceps tubercle (= acrocoracoid process): absent or poorly developed (0), conspicuous and well developed as tuber (1), developed as a posteroventrally oriented ridge (2).
228. Coracoid, prominent fossa on ventral surface posteroventral to glenoid (subglenoid fossa): absent (0); present (1).
229. Humerus, shape of head: elongate (0), globular (1).
230. Humerus, longitudinal torsion of shaft: absent (0), present (1).
231. Humerus, size of trochanters relative to midshaft diameter: < (0), > 150% (1) > 250% (2). ordered
232. Humerus, development of internal tuberosity: low/rounded (0), hypertrophied (1).
233. Humerus, length of deltopectoral crest relative to total bone length: < 0.4 (0), 0.43–0.49 (1) > 0.52 (2). ordered
234. Humerus, development of deltopectoral crest: large rectangular crest (0), reduced to a low, rounded flange (1).
235. Humerus, orientation of deltopectoral crest apex: anteriorly (0), anterolaterally (1).
236. Humerus, relative orientation of proximal & distal condyles in anteroposterior view: parallel, humerus straight (0), distal canted (1).
237. Humerus, anterior surface of bone adjacent to ulnar condyle: smooth or gently depressed (0), bears well-defined fossa (1).
238. Humerus, shape of distal condyles: rounded (0), flattened (1).
239. Radius and ulna, development of radial external tuberosity and ulnar internal tuberosity: low, rounded (0), hypertrophied distal ends of radius and ulna broadened (1).
240. Radius, shaft: straight (0); curves laterally (1).
241. Radius, development of medial biceps tubercle: small or indistinct (0), hypertrophied (1).
242. Ulna, olecranon process: absent (0), present (1).
243. Ulna, morphology of olecranon process: transversely robust (0); transversely compressed and ‘blade-like’ (1).
244. Ulna, crest extending distally along posterior surface from olecranon process: absent (0), present (1).
245. Ulna, hypertrophied medial and lateral processes on proximal end: absent (0), present (1).
246. Ulna, length relative to minimum circumference: stout, < 2.3 (0); gracile > 2.6 (1).
247. Carpus, morphology and articulations of distal carpals: separate dc1 and dc2 over separate metacarpals, flattened proximodistally (0), fused dc1 and dc2, dc1 overlaps metacarpals I and II, flattened proximodistally (1), fused dc1 and dc2, dc1 overlaps metacarpals I and II, strongly arched proximodistally (2).
248. Manus, length of digit II relative to length of humerus: < (0), > (1) (modified from Carrano et al. (*2*); Rauhut et al.(*4*))
249. Manus, composition: digit IV and V present (0), digit IV present, digit V absent (1), MC IV present, IV phalanges and digit V absent (2), digits IV and V absent (3). ordered
250. Manual digits, lengths: III longest (0), II longest (1).
251. Metacarpals, transverse width of proximal articular ends relative to minimum transverse shaft width: < (0), ≥ 2x (1).
252. Metacarpal I, length to minimum width ratio: 1.4–1.9 (0), ≥ 2.4 (1).
253. Metacarpal I, length relative to length of metacarpal II: > 57% (0), < 57% (1).
254. Metacarpal I, extent of contact with metacarpal II relative to shaft length: < 1/3 (0), 1/2 (1).
255. Metacarpal I, angle between facet for metacarpal II and proximal articular facet: perpendicular (0), obtuse (1).
256. Metacarpal III, position of base relative to those of other metacarpals: at same level (0), on palmar surface (1).
257. Metacarpal III, shape of proximal end: rectangular (0), triangular (1).
258. Metacarpal III, width relative to width of metacarpal II: > 50% (0), < 50% (1).
259. Manual ungual I, length:height ratio: < 2.5x (0), > 2.5x (1).
260. Manual unguals, proximal height:width ratio: transversely broad, < 2.0 (0), transversely narrow, > 2.4 (1).
261. Pelvic elements, articulations in adults: separate (0), fused (1).
262. Ilium, large external pneumatic foramina and internal spaces: absent (0), present (1).
263. Ilium, vertical ridge on lateral surface of blade dorsal to acetabulum: absent (0), low swollen ridge (1), low double ridge (2).
264. Ilium, posterior width of brevis fossa: subequal to anterior width, fossa margins subparallel (0), twice anterior width, fossa widens posteriorly (1).
265. Ilium, height of lateral wall of brevis fossa relative to medial wall: taller along whole length (0), shorter anteriorly, exposing medial wall in lateral view (1).
266. Ilium, morphology between supraacetabular crest and brevis shelf on lateral surface: gap (0), continuous ridge (1).
267. Ilium, ventrolateral development of supraacetabular crest: large/pendant ‘hood’ (0), reduced shelf (1).
268. Ilium, orientation of pubic peduncle: mostly ventral (0), mostly anterior or ‘kinked’ double facet with anterior and ventral components (1).
269. Ilium, shape of acetabular margin of pubic peduncle: transversely convex or flat (0); transversely concave (1).
270. Ilium, relative sizes of pubic and ischial articulations: subequal (0), pubic articulation ≥ 130% of iliac articulation (1).
271. Ilium, morphology of ischial peduncle: rounded (0), acuminate (1).
272. Ilium, pubic peduncle length to width ratio: ≤ 1 (0), 1.3–1.75 (1), > 2 (2). ordered
273. Ilium, ridge on medial surface adjacent to preacetabular notch: absent (0), present (1), strongly developed, forming a shelf (2). ordered
274. Ilium, preacetabulum length relative to anterior edge of pubic peduncle: reaches anteriorly to same point as (‘brachyiliac’) (0), or well past (‘dolichoiliac’) (1).
275. Ilium, depth of preacetabular process: shallow (0), deep (1).
276. Ilium, anteroventral lobe of preacetabular process: absent (0), present (1).
277. Ilium, shape of dorsal margin: convex (0), straight (1).
278. Ilium, postacetabulum length relative to ischial peduncle length: ≤ (0), > (1).
279. Ilium, depth of postacetabular process: shallow (0), deep (1).
280. Shape of posterior margin of postacetabular process of ilium: rounded, dorsal margin curves gradually into posterior margin (0), only slightly convex or straight, dorsal margin offset from posterior margin by marked posterodorsal angle (1), concave (2), tapering (3). (modified from Carrano et al.^2^ and Rauhut et al.^4^)
281. Posterior process on the dorsal end of the posterior margin of the postacetabular blade, formed by a posterior expandsion of the medial brevis shelf: absent (0), present (1). (modified from Carrano et al.^2^ and Rauhut et al.^4^)
282. Obturator foramen in pubis: completely enclosed in bone (0), ventrally open notch (1), absent (2) ordered
283. Pubic fenestra below obturator foramen: absent (0); present (1)
284. Obturator plate of ischium: continuous with pubic articulation and without foramen (0), with large foramen below the pubic peduncle (1), with large notch below the pubic peduncle, obturator process offset from pubic articulation (2)
285. Pubis, shaft orientation: straight (0), ventrally curved (1).
286. Pubis, articulation between apices in adults: unfused (0); fused (1).
287. Pubis, contact between distal portions: separate distally (0), contacting (1), contacting with slit-like opening proximal to distal expansion (interpubic fenestra) (2).
288. Pubis, angle between long axes of shaft and boot: 75–90° (0), < 60° (1).
289. Pubis, morphology of symphysis: marginal (0), broad (1).
290. Pubis, morphology of obturator foramen: small and subcircular (0), large and oval (1).
291. Pubis, anterior expansion of distal end: absent (0), present (1).
292. Pubis, boot length relative to shaft length: < (0), > 30% (1), > 60% (2). ordered
293. Pubis, shape of boot in ventral view: broadly triangular (0), narrow, with subparallel margins (1).
294. Pubis, articulation with ilium: planoconcave (0), peg-and-socket (1).
295. Ischium, length relative to pubis length: 75–80% (0), ≤ 70% (1), > 80% (2).
296. Ischium, shaft orientation: straight (0), ventrally curved (1).
297. Ischium, articulation with ilium: planoconcave (0), peg-and-socket (1).
298. Ischium, morphology of antitrochanter: large and notched (0), reduced (1).
299. Ischium, notch ventral to obturator process: absent (0), present (1).
300. Ischium, morphology of symphysis: unexpanded (0), expanded as apron (1).
301. Ischium, cross-sectional shape of paired midshafts: oval (0), heart-shaped, medial portions of shafts extend posteriorly as midline flange (1).
302. Ischium, morphology of distal end: rounded (0), expanded, triangular (1).
303. Ischium, articulation at distal end in adults: separate (0), fused (1).
304. Femur, head orientation: 45° anteromedial (0), 10–30° anteromedial (1), medial (2). ordered
305. Femur, head angle: ventromedial (0), horizontal (medial) (1), dorsomedial (2). ordered
306. Femur, groove on proximal surface of head oriented oblique to long axis of head (‘articular groove’): absent (0), present (1).
307. Femur, oblique ligament groove on posterior surface of head: shallow, groove bounding lip does not extend past posterior surface of head (0), deep, bound medially by well-developed posterior lip (1).
308. Femur, placement of lesser trochanter relative to femoral head: does not reach ventral margin (0), rises past ventral margin (1), rises to proximal surface (2). ordered
309. Femur, morphology of anterolateral muscle attachments at proximal end: continuous trochanteric shelf (0), distinct lesser trochanter and attachment bulge (1).
310. Femur, development of fourth trochanter: prominent semioval flange (0), very weak or absent (1).
311. Femur, distinctly projecting accessory trochanter (derived from lesser trochanter): weak, forms slightly thickened margin of lesser trochanter (0), present as triangular flange (1).
312. Femur, M. femorotibialis externus origin medially on anterodistal surface: faint, small rugose patch (0), pronounced rugose depression that extends to distal femur (1).
313. Femur, development of medial epicondyle: rounded (0), ridge (1).
314. Femur, distal extensor groove: absent (0), present (1).
315. Femur, morphology and orientation of tibiofibularis crest: broad (0), narrow, longitudinal (1), lobular, oblique (2).
316. Femur, infrapopliteal ridge connecting medial distal condyle and crista tibiofibularis: absent (0), present (1).
317. Femur, orientation of long axis of medial condyle in distal view: anteroposterior (0), posterolateral (1).
318. Femur, projection of lateral and medial distal condyles: approximately equal (0), lateral projects distinctly further than medial, distal surface of medial is gently flattened (1).
319. Femur, morphology of distal end: central depression connected to crista tibiofibularis by a narrow groove (0), anteroposteriorly oriented shallow trough separating medial and lateral convexities (1).
320. Tibia, lateral malleolus: backs astragalus (0), overlaps calcaneum (1).
321. Tibia, shape of edge of lateral malleolus: smoothly curved (0), tabular notch (1).
322. Tibia, morphology of distal cnemial process: rounded (0), expanded proximodistally (1).
323. Tibia, morphology of lateral (fibular) condyle: large (0), small and lobular (1).
324. Tibia, anterolateral process of lateral condyle: absent or horizontal projection (0), prominent, curves ventrally (1).
325. Tibia, anteromedial buttress for astragalus: absent (0), ventral (1), marked oblique step-like ridge (2), reduced oblique ridge (3), bluntly rounded vertical ridge on medial side (4).
326. Tibia, morphology of fibular crest: narrow (0), bulbous (1).
327. Tibia, development of fibular crest: extends to proximal end of tibia as high crest (0), extends to proximal end of tibia as low ridge (1), does not extend to proximal end of tibia (2). ordered
328. Groove or depression on the medial side of the proximal end of the fibula: absent or only shallow concavity present (0), deep groove on the posterior half of the medial side of the proximal end, offset from anterior margin and opening at least partially posteromedially (1), large, deep depression that opens medially and is offset from the anterior margin only by a thin ridge or lip (2)
329. Ridge on the medial side of the proximal end of the fibula that extends anterodistally from the posteroproximal part: absent (0), present (1).
330. Fibula, size of iliofibularis tubercle: faint scar (0), large (1), anterolaterally curving flange (2).
331. Fibula, size of proximal end relative to width of proximal tibia: < 75% (0), ≥ 75% (1).
332. Astragalus, articulation between ascending process and fibula in adults: separate (0), fused (1).
333. Astragalus, orientation of distal condyles: ventral (0), 30-45° anterior (1).
334. Astragalus, ascending process morphology: blocky (0), laminar (1).
335. Astragalus, angle of dorsal margin of ascending process: low and oblique (0), high and oblique (1).
336. Astragalus, ascending process height relative to depth of astragalar body: less (0), subequal (1), > 1.6 times (2).
337. Astragalus, prominent proximolateral extension: absent (0); present (1).
338. Astragalus, round fossa at base of ascending process: absent (0), small (1), large (2). ordered
339. Astragalus, development of articular surface for distal end of fibula: large, dorsal (0), reduced, lateral (1).
340. Astragalus, posterolateral crest: absent (0), present (1).
341. Astragalus, posteromedial crest: absent (0), present (1).
342. Astragalus, articulation with calcaneum in adults: separate (0), fused (1).
343. Metatarsal I, length relative to length of metatarsal II: ≥ 50% (0), < 50% (1).
344. Metatarsal III, shape of proximal end: rectangular (0), shallow notch (1), deep notch (2). ordered
345. Metatarsal III, midshaft cross-sectional shape: rectangular (0), wedge-shaped, plantar surface pinched (1).
346. Metatarsal III, relative proportions of shaft: short and thick, length:transverse width ratio < 12.0 (0), long and gracile, ratio > 12.5 (1).
347. Metatarsal IV, proportions of distal end: broader than tall (0), taller than broad (1).
348. Metatarsal V, morphology of distal end: articular (0), non-articular (1).
349. Metatarsal V, length relative to length of metatarsal IV: > 50% (0), < 50% (1).
350. Antarctometatarsus: absent (0), present (1).
351. Pedal unguals, morphology of lateral and medial grooves: single (0), double (1).
352. Pedal unguals, digits III and IV, cross-sectional shape: triangular (0), elliptical (0).
353. Pedal unguals, digit II, mediolateral symmetry: symmetrical (0), asymmetrical (1).
354. Pedal digit phalanges, length of I-1 + I-2 relative to III-1: greater (0), less than or equal
355. Neural spine of third cervical vertebra: not significantly different from other postaxial cervical neural spines (0), slender and strongly backswept (1) (New)

**Analysis of homoplasy**

Measuring homoplasy concentration on a tree

We introduce here a new measure, homoplasy concentration (HC), devised to quantify the concentration of homoplasy on a phylogenetic tree. As homoplasy tends to be more problematic for phylogenetic inference if it is concentrated on nodes or branches that are proximal to the node in question, we incorporated this information in the new measure. The measure is based on counting the distance (in terms of the number of nodes) between homoplastic transformations that lead to the same character state (convergences, parallelisms) or reversals from and to the same character state. For a given branch (*b*) that shows a step in a given character (*i)*, the measure HC is:


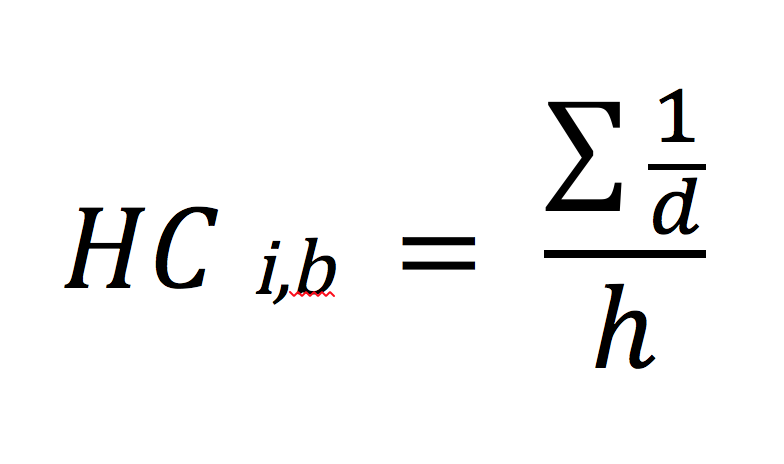


where *d* is the distance in number of nodes to the homoplastic step/s that exist on the tree (in which the same apomorphic character state is acquired), and h is the number of homoplastic steps on the tree (that result in the acquisition of the same character state). This HC will produce high values when there are many homoplastic steps in nearby nodes and low values when there is no homoplasy or when homoplastic steps are optimized on the tree in distant nodes.

Ambiguous optimizations are common in parsimony analyses, especially with fossils that introduce missing data and uncertainty regarding the placement of character transformations on the tree. In the cases of ambiguous optimizations we iteratively evaluate all possible most parsimonious reconstructions and for each branch we add the nodal distance of homoplastic step/s in each reconstruction and divide by the sum of homoplastic step/s across all possible reconstructions.

Once this measure is calculated for each character and each branch, it is summed across all characters to obtain the HC value for each branch:


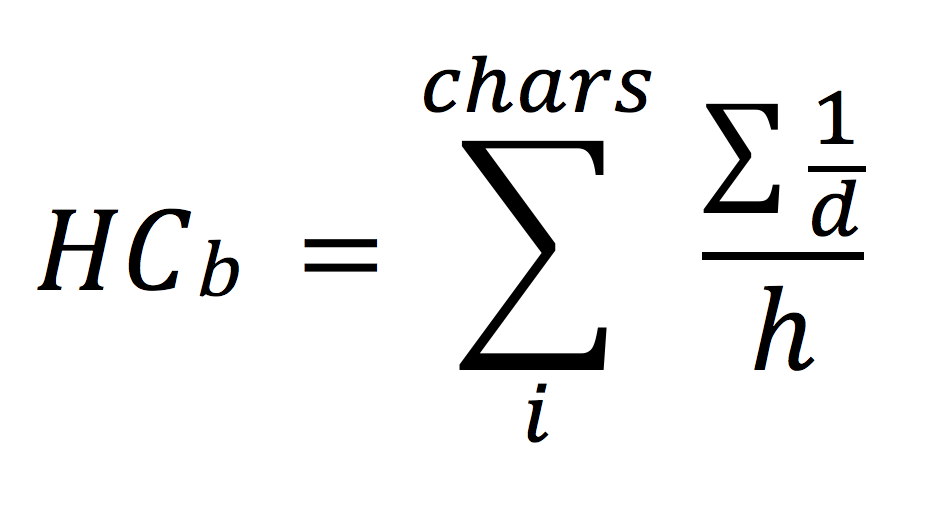


The results how a concentration of high values of HC close to the radiation of Tentanurae. Below we show the distribution of HC values plotted against the nodal distance to the clade Tetanurae. The HC values of branches are negatively correlated with their distance to this node.


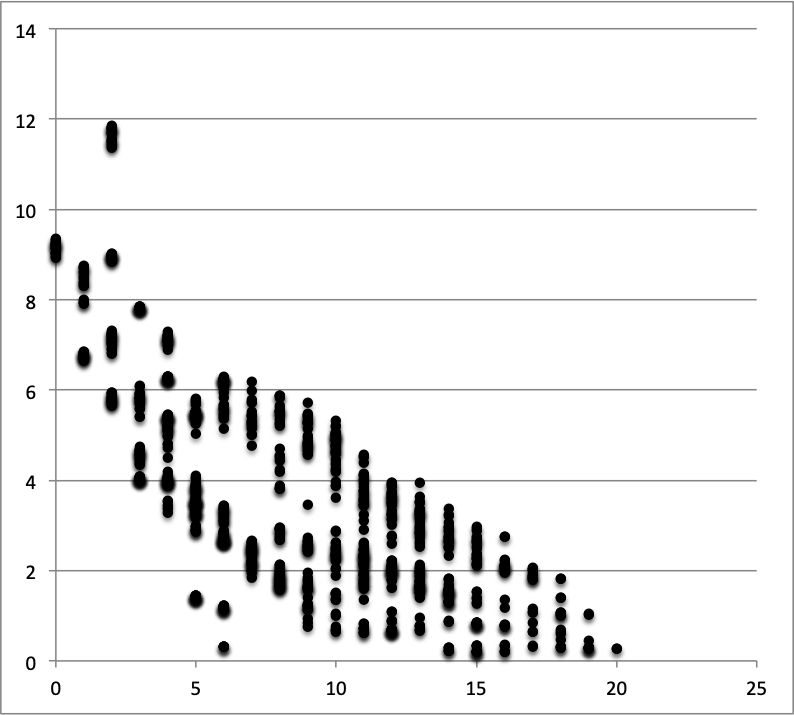


Displaying HC values on a tree

The measure HC is calculated and a numerical value is obtained for each branch of the tree. To show these values on a tree we use a color code from blue (HC=0) that represents the absence of homoplastic steps in that branch (for all characters) to red (HC= maximal HC value obtained for the tree) that represents concentration of homoplasy in nearby nodes. As the HC values are dependent on the homoplasy levels and the number of characters, the color-coding is devised to compare the branches of the tree within the context of the same data matrix (not among different trees derived from different datasets). Note that branches colored in close to blue values may have either very low numbers of characters with homoplastic steps or multiple characters showing homoplastic changes but those have the convergent homoplasies located in distant nodes (homoplasy not concentrated).

Below we show one of the MPTs with color-coded branches according to their HC values. Length of the branches represents the calibration of times of divergence based on FADs of each taxon.


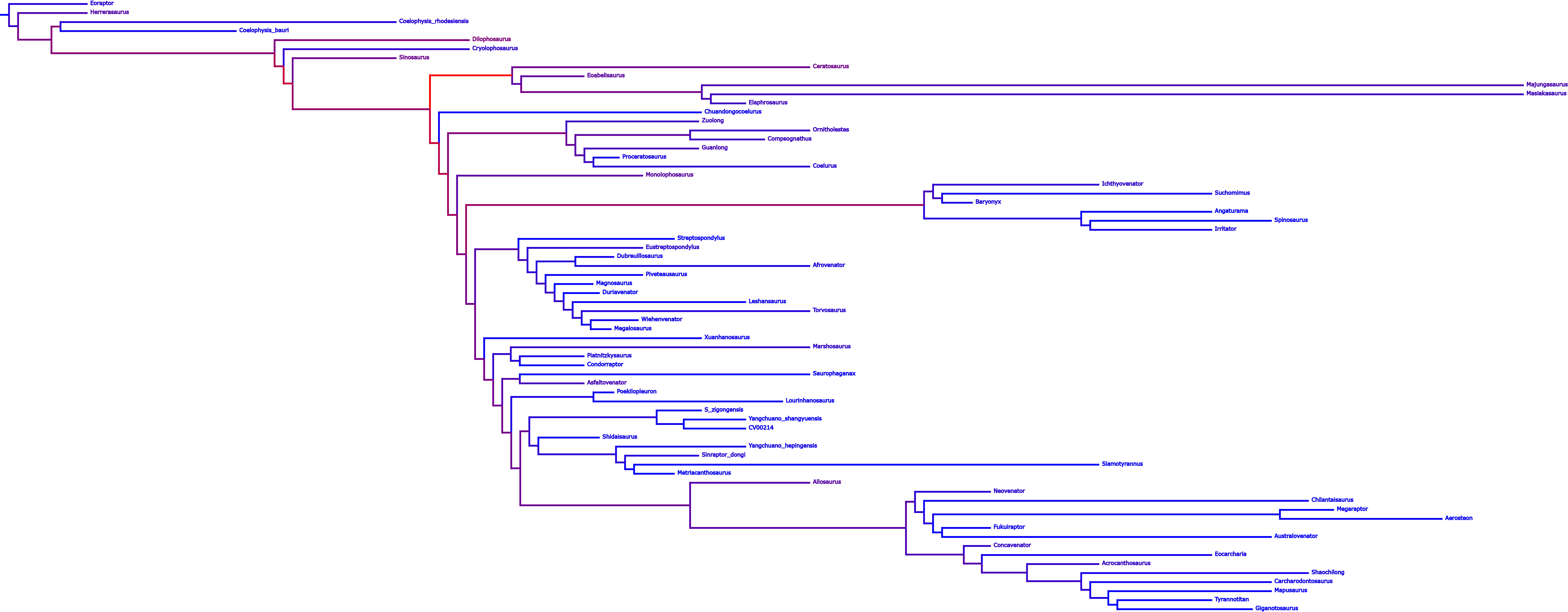


Integrating Homoplasy Concentration across all trees

The measure HC is calculated for each most parsimonious tree (MPT). To summarize the results we identify the equivalent branches for all the MPTs and average their HC values to plot the average HC in the consensus tree (either strict consensus or reduced strict consensus).

Evaluating concentration of homoplasy across time

Certain evolutionary episodes (e.g., radiations) imply the appearance of multiple branches in a relatively short amount of time. Here we use parsimony optimization and trees calibrated with the chronostratigraphic age of each terminal taxon to test the distribution of changes (and also the homoplastic changes) across time. For the calibration of trees we used a fixed minimum branch length of 1 million years for zero-length temporal branches.

In order to obtain the distribution of morphological changes through time we count, for a given branch of the tree, the number of characters showing changes and divide this number by the temporal duration of the branch. In the case of ambiguous optimizations we iterate through alternative reconstructions to obtain the average count of changes along that branch in all possible reconstructions. Note that by dividing our estimates of changes by the temporal duration of the branch we are assuming an even distribution of changes through time *within* each branch (not between different branches). For a given time bin one branch can have a high number of changes per million years and other branch can have no changes at all.

In our study we use time bins of 1 million years and we average the number of changes per million years across all branches that go through a given time bin. Thus, if there are two branches going through a given time bin and one has an estimate of 2 changes (per million years) and the other had no changes, we will obtain an average of 1 change in that time bin. Using the average rather than the sum also aims to correct for uneven number of branches for different time bins.

The distribution of changes through time shows a peak of concentrated character change during the radiation of Tetanurae, compatible with the occurrence of a high rate of morphological evolution during this radiation. The following graph shows the distribution of morphological changes per unit of time, with geological age along the x-axis and the average number of character changing along the y-axis. A first narrow peak is present along basal nodes of Neotheropoda in the Late Triassic (200-205 Ma) and a broader peak during the radiation of Tetanurae (and Ceratosauria) close to the Early-Middle Jurassic boundary.


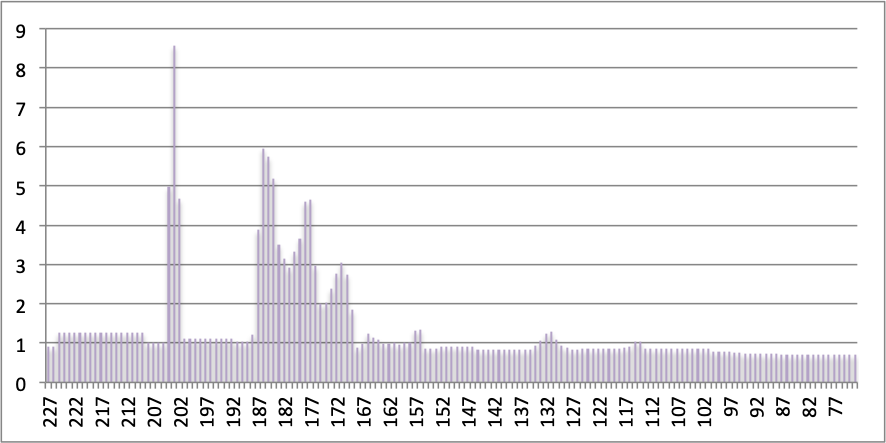


A similar approach was taken for estimating the distribution of homoplasy through time, by counting character transformation only if they show homoplastic changes in other branches of the tree that lead to the same character state (convergences, parallelisms) or reversals from and to the same character state. The graph below shows the distribution of homoplastic steps through time, which mimics the one above on the rate of morphological change. This shows the elevated rates of morphological change across the late Early Jurassic and Middle Jurassic explains the elevated levels of homoplasy along the branches that radiated at that time.


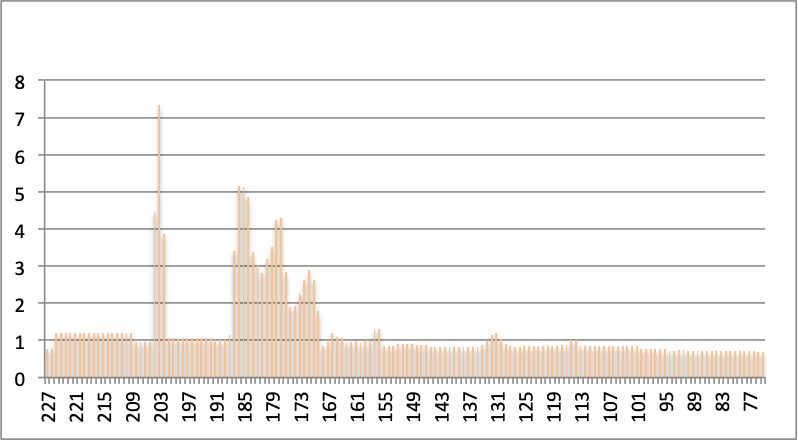


Implementation in TNT

The entire procedure described above is integrated above is integrated in a script for TNT (phomoplasy.run) that takes as input the data matrix, the set of trees to analyse (either the MPTs alone or the MPTs followed by the consensus that will be used to summarize the results), and the first appearance datum (FAD) and last appearance datum (LAD) age of each terminal taxon (detailed in a text file named FALAD).


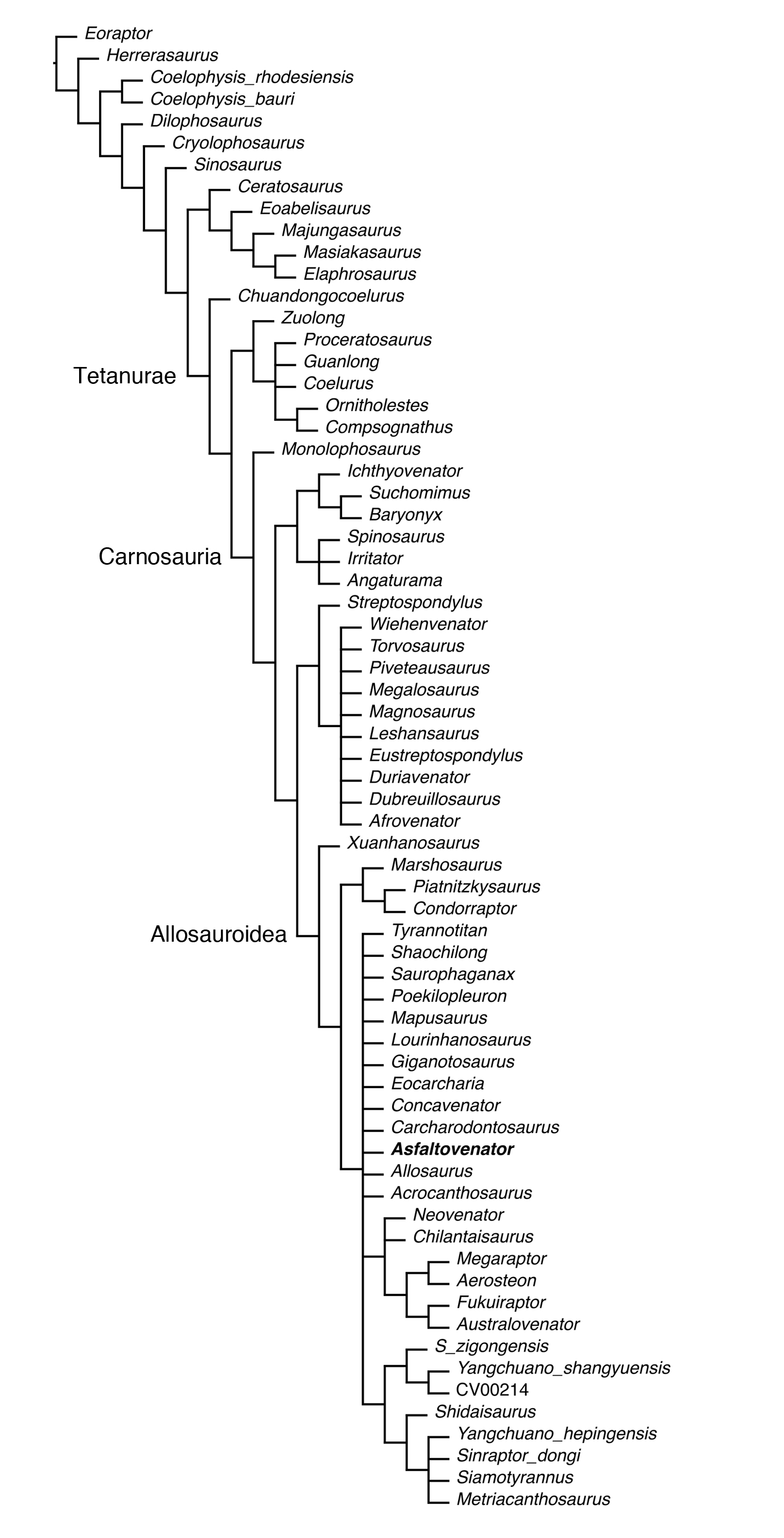


Fig. S1.

Strict consensus tree of phylogenetic analysis.


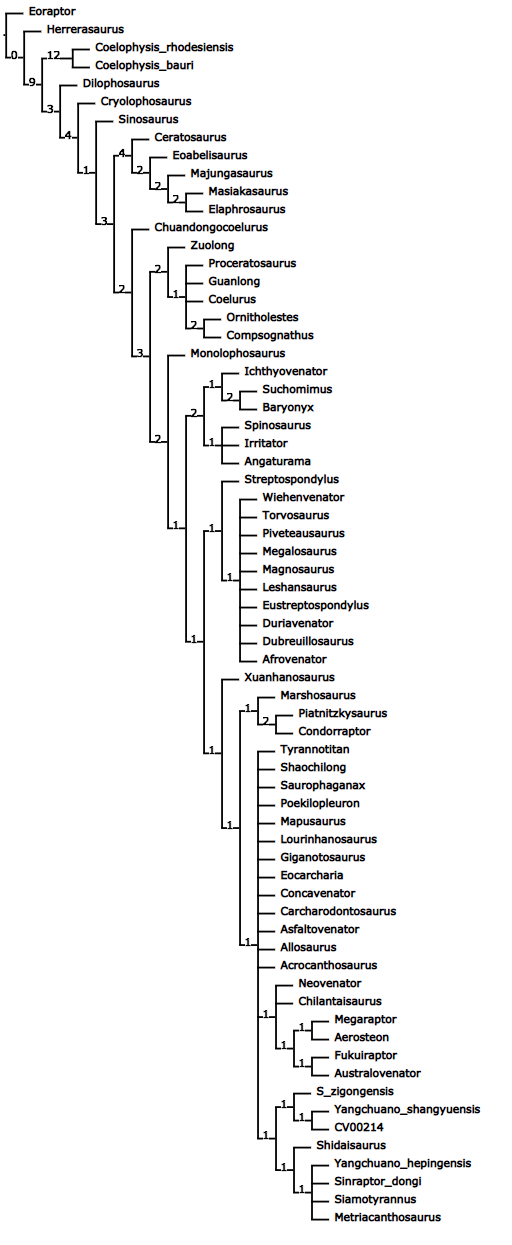

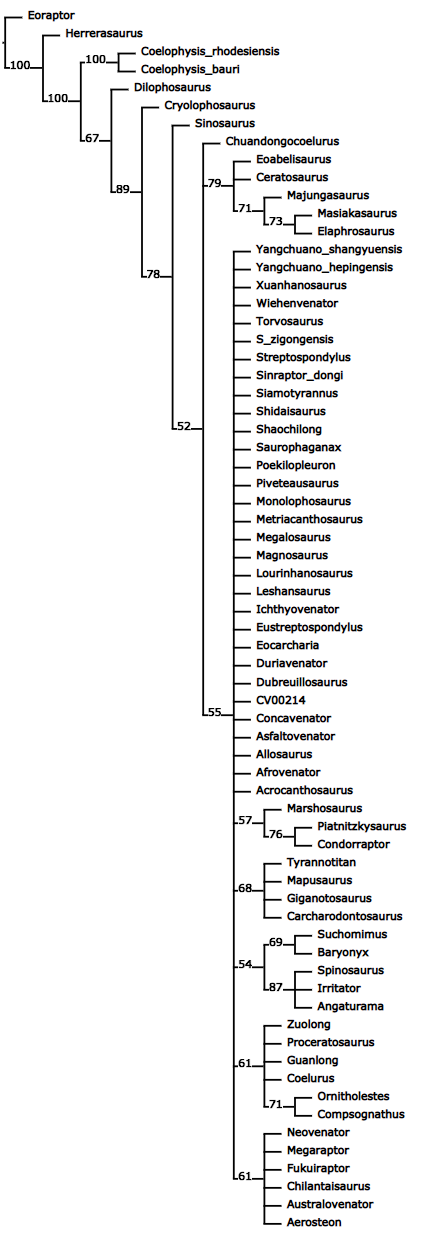


Fig. S2.

Bremer support (left) and bootstrap values (right) of nodes of phylogenetic analysis.


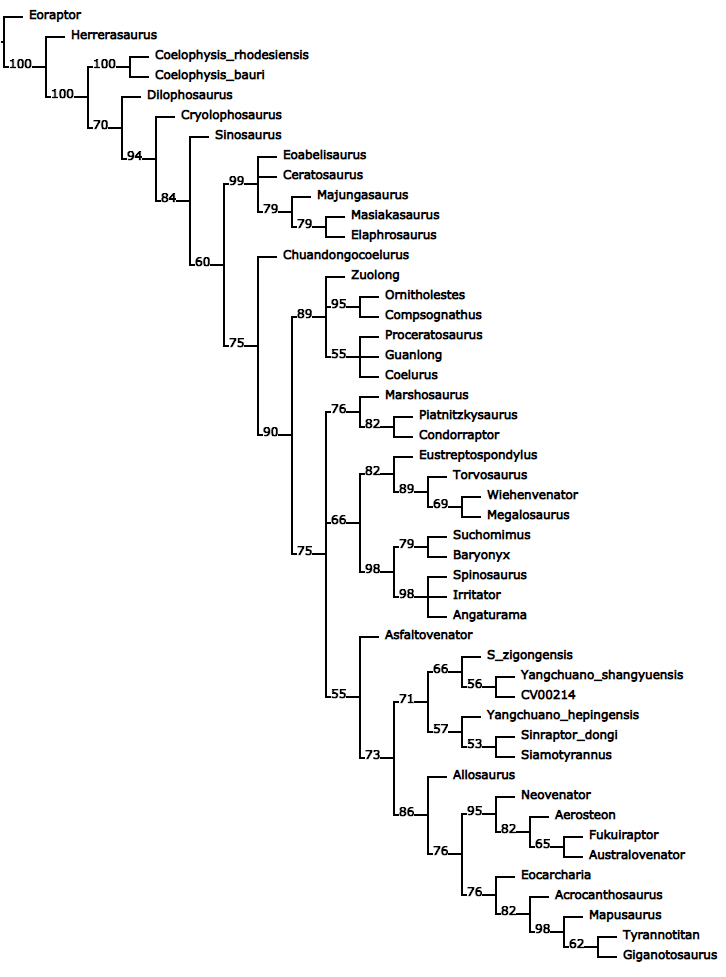

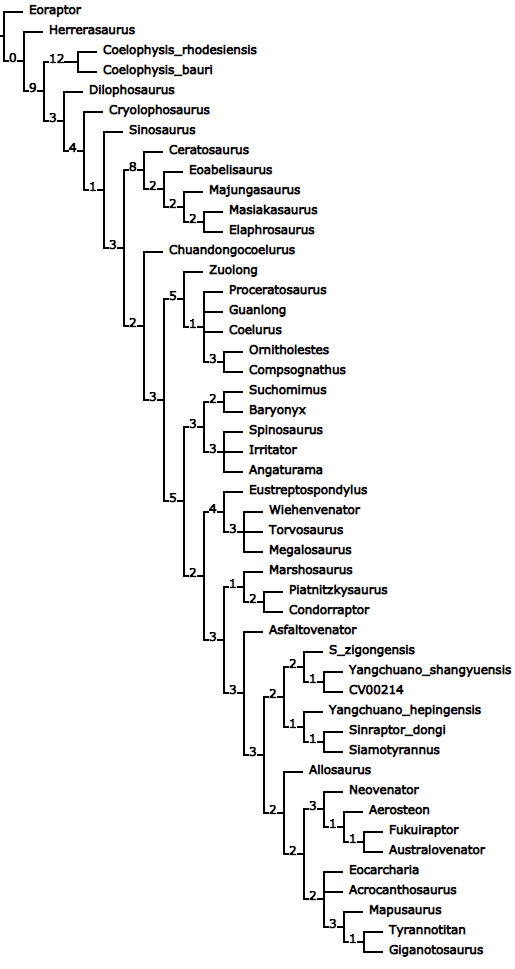


Fig. S3.

Bremer support (left) and jackknive values (right) for trees excluding particularly unstable taxa.


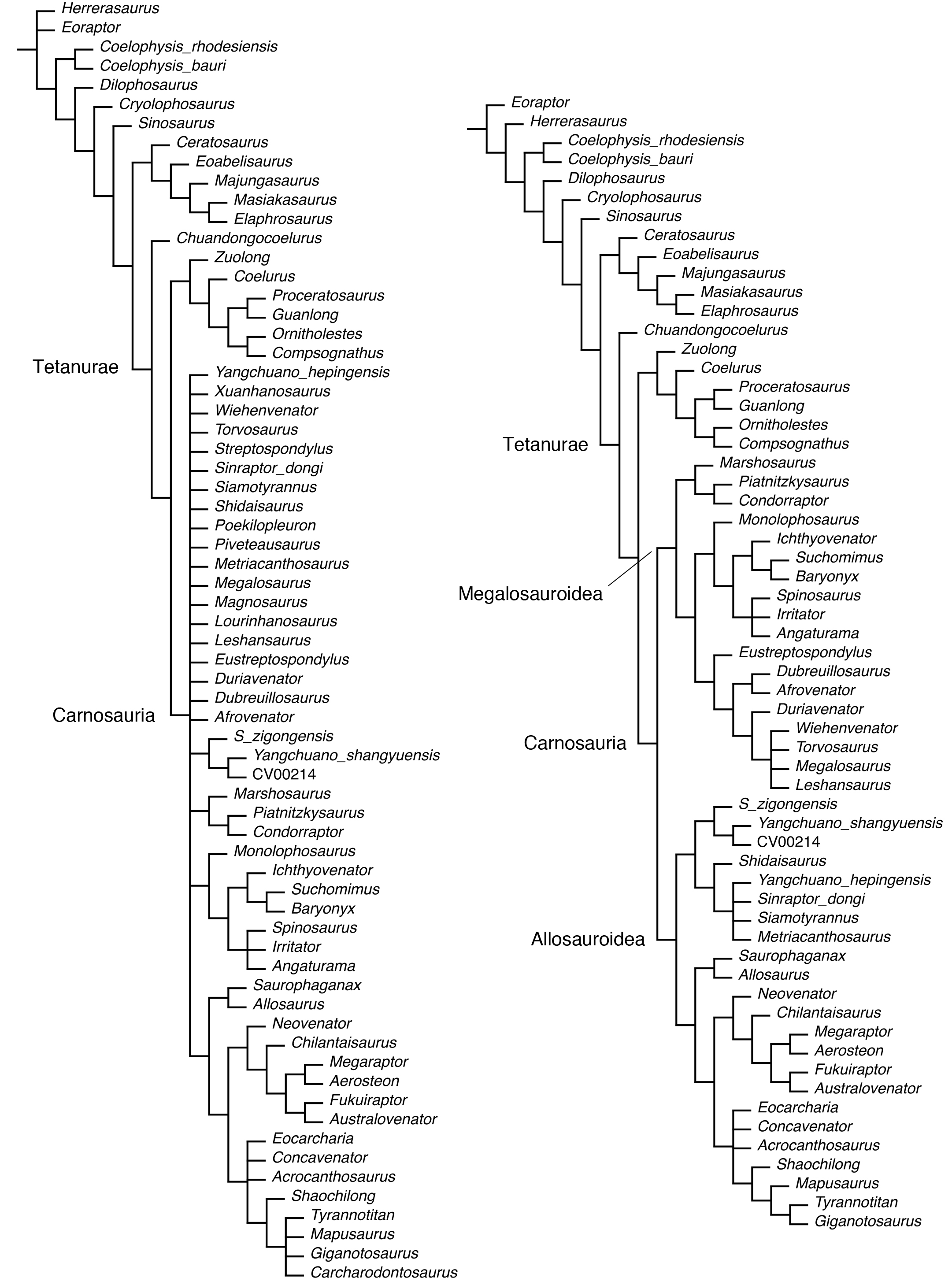


Fig. S4.

Strict and reduced consensus tree of phylogenetic analysis excluding *Asfaltovenator*.


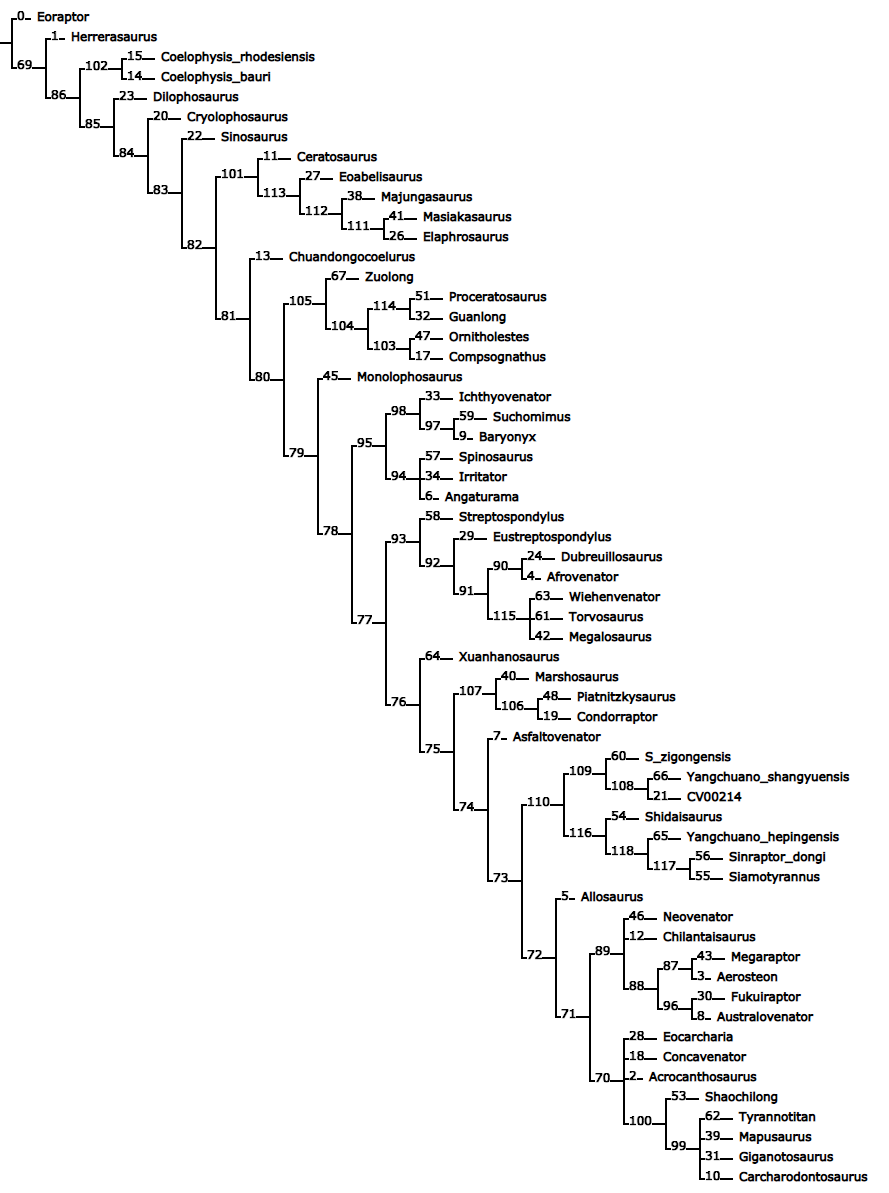


Fig. S5.

Reduced consensus tree with labelled nodes.


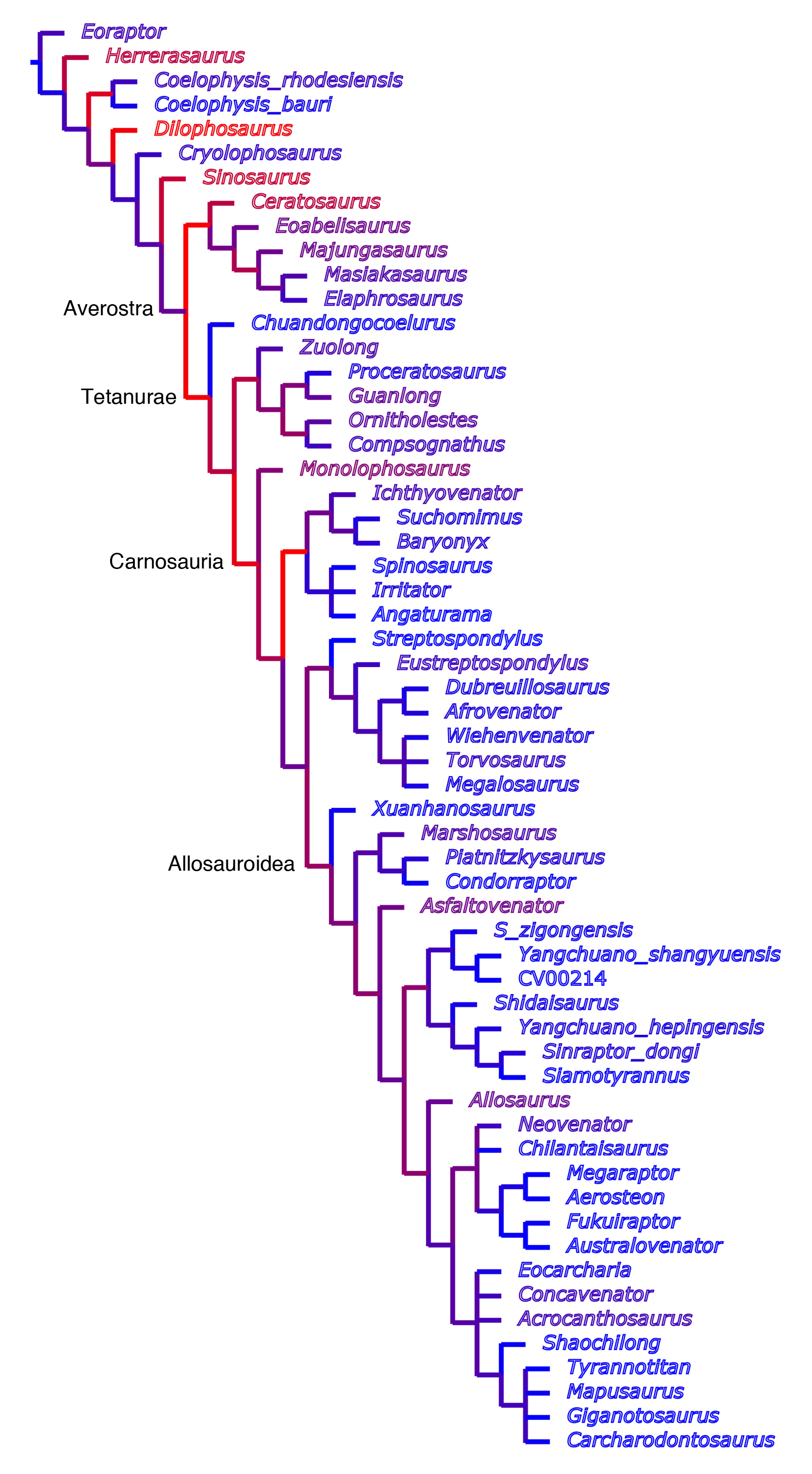


Fig. S6.

Colour-coded homoplasy distribution mapped on the reduced consensus tree.

| **Element** | **length** | **twp** | **dp** | **twd** | **dd** | **msw** | **msc** | **others** |
| --- | --- | --- | --- | --- | --- | --- | --- | --- |
| **humerus dex.** | 335 | 140 | 70 | 111 | 56 | c. 50 | c. 153 | ldpc. 148 |
| **humerus sin.** | 343 | 144 | 65 | 110 est. | 53 | c. 53 | c. 155 | ldpc. 148 |
| **ulna dex.** | 250 | 66 | 105 | 57 | 39 | 25 | c. 93 | w/o ol 205 |
| **ulna sin.** | 232 | 68 | 103 | 53 | 40 | 27 | c. 95 | w/o ol 202 |
| **radius dex.** | c. 200 | 38 | 63 | 46 | 47 | c. 26 | c. 100 |  |
| **radius sin.** | c. 205 | 39 | 63 | 48 | 55 | — | — |  |
| **carpus** | 35 |  |  |  |  |  |  | wmc c. 110 |
| **Mc I dex.** | 59 | 39 | 40 | 43 | 43 | 40 |  | minl: 43 |
| **Mc II dex.** | 109 | 62 | 50 | 48 | 48 | 26 |  |  |
| **Mc III dex.** | 95 | 33 | 42 | 23 | 28 | 10 |  |  |
| **P I-1 dex.** | 112 | 41 | c. 55 | 40 | 37 | 30 |  |  |
| **P I-2 dex.** | c. 97 | 36 | 70 |  |  |  |  | maxl c. 150 |
| **P II-1 dex.** | 77 | 42 | 46 | 32 | 33 | 25 |  |  |
| **P II-2 dex.** | 83 | 30 | 43 | 30 | 31 | 22 |  |  |
| **P II-3 dex.** | — | 27 | 57 |  |  |  |  |  |
| **P III-1 dex.** | 32 | 19 | 29 | 16 | 19 | 9 |  |  |
| **P III-2 dex.** | c. 28 | 11 | 14 | 9 est. | 9 est. | 9 |  |  |
| **P III-3 dex.** | 39 | 10 | 16 | 11 | 18 | 7 |  |  |
| **P III-4 dex.** | 53 est. | 15 | 32 |  |  |  |  | maxl 55 est. |
| **digit I** | c. 185 |  |  |  |  |  |  |  |
| **digit II** | c. 220 |  |  |  |  |  |  |  |
| **digit III** | c. 145 |  |  |  |  |  |  |  |
| **P II-3 sin.** | 77 | 28 | 57 |  |  |  |  | maxl 107 |

**Table S1.**

Measurements of forelimb elements of *Asfaltovenator*. Length of unguals was measured perpendicular to the proximal articular facet. dex, dextrum (right); dd, depth distally; dp, depth proximally; est., estimated; ldpc, length to end of deltopectoral crest; maxl, maximal length; Mc, metacarpal; minl, minimal length; msc, minimal shaft circumference; msw, minimal shaft width; P, phalanx; twd, transverse width distally; twp, transverse width proximally; wmc, width of articulated metacarpus; w/o ol, without olecranon process.

| **Taxon** | **AFA** | **ALA** | **Taxon** | **AFA** | **ALA** |
| --- | --- | --- | --- | --- | --- |
| *Eoraptor* | 231.7 | 225 | *Irritator* | 113 | 100.5 |
| *Herrerasaurus* | 231.7 | 225 | *Leshansaurus* | 158 | 152.1 |
| *Acrocanthosaurus* | 120 | 113 | *Lourinhanosaurus* | 155 | 148 |
| *Aerosteon* | 83.6 | 75 | *Magnosaurus* | 170.3 | 169 |
| *Afrovenator* | 170 | 145 | *Majungasaurus* | 72.1 | 66 |
| *Allosaurus* | 157.3 | 145 | *Mapusaurus* | 96 | 93.9 |
| *Angaturama* | 113 | 100.5 | *Marshosaurus* | 157.3 | 145 |
| *Asfaltovenator* | 176.15 | 170 | *Masiakasaurus* | 72.1 | 66 |
| *Australovenator* | 97 | 93.9 | *Megalosaurus* | 168.3 | 167 |
| *Baryonyx* | 129.4 | 127 | *Megaraptor* | 92 | 87 |
| *Carcharodontosaurus* | 113 | 93.9 | *Metriacanthosaurus* | 163.5 | 160 |
| *Ceratosaurus* | 157.3 | 145 | *Monolophosaurus* | 166.1 | 163.5 |
| *Chilantaisaurus* | 93.9 | 89.8 | *Neovenator* | 129.4 | 125 |
| *Chuandongocoelurus* | 161 | 157 | *Ornitholestes* | 157.3 | 145 |
| *Coelophysis bauri* | 227 | 208.5 | *Piatnitzkysaurus* | 176.15 | 170 |
| *C. rhodesiensis* | 201.3 | 190.8 | *Piveteausaurus* | 165 | 163.5 |
| *Coelurus* | 157.3 | 145 | *Poekilopleuron* | 168 | 166.7 |
| *Compsognathus* | 154 | 150 | *Proceratosaurus* | 168 | 166.1 |
| *Concavenator* | 127 | 125 | *Saurophaganax* | 157.3 | 145 |
| *Condorraptor* | 176.15 | 170 | *Shaochilong* | 93.9 | 89.8 |
| *Cryolophosaurus* | 199.3 | 182.7 | *Shidaisaurus* | 174.1 | 168.3 |
| CV 00214 | 158 | 152.1 | *Siamotyrannus* | 129.4 | 113 |
| *Sinosaurus* | 201.3 | 190.8 | *Sinraptor* | 163.5 | 157.3 |
| *Dilophosaurus* | 199.3 | 182.7 | *Spinosaurus* | 100.5 | 93.9 |
| *Dubreuillosaurus* | 168 | 166.7 | *Streptospondylus* | 165 | 160 |
| *Duriavenator* | 169.5 | 168.3 | *Suchomimus* | 125 | 100.5 |
| *Elaphrosaurus* | 155 | 152.1 | *"S." zigongensis* | 161 | 157 |
| *Eocarcharia* | 125 | 100.5 | *Torvosaurus* | 157.3 | 145 |
| *Eustreptospondylus* | 165 | 163.5 | *Tyrannotitan* | 110 | 100.5 |
| *Fukuiraptor* | 129.4 | 125 | *Wiehenvenator* | 165 | 164 |
| *Giganotosaurus* | 100.5 | 96 | *Xuanhanosaurus* | 161 | 157 |
| *Guanlong* | 163.5 | 157.3 | *Yangchuanosaurus* | 158 | 152.1 |
| *Ichthyovenator* | 125 | 113 | *Zuolong* | 163.5 | 157.3 |

Table S2.

Ages assigned to taxa for time calibrated cladograms and anaylsis of homoplasy over time bins. Ages of taxa from the Cañadón Asfalto Formation, including *Asfaltovenator*, are based on Cúneo et al.^9^ and Figari et al^58^. Ages for the taxa of the Lower and Upper Shaximiao Formations, which are often said to be Middle Jurassic in age^4^, are based on the recent re-evaluation of the age of the Lower Shaximiao Formation by Wang et al^59^. AFA, age of first appearance; ALA, age of last appearance.

**Supplementary references**

1. Bonaparte, J. F. Dinosaurs: a Jurassic assemblage from Patagonia. *Science* **205**, 1377-1379 (1979).
2. Ostrom, J. H. in *Aspects of vertebrate history* (ed L. L. Jacobs) 245-256 (Museum of Northern Arizona Press, 1980).
3. Carpenter, K., Miles, C., Ostrom, J. H. & Cloward, K. in *The carnivorous dinosaurs* (ed Kenneth Carpenter) 49-71 (Indiana University Press, 2005).
4. Choiniere, J. N., Clark, J. M., Forster, C. A. & Xu, X. A basal coelurosaur (Dinosauria: Theropoda) from the Late Jurassic (Oxfordian) of the Shishugou Formation in Wucaiwan, People's Republic of China. *J. Vert. Paleont.* **30**, 1773-1796 (2010).
5. Ezcurra, M. D. & Cuny, G. The coelophysoid *Lophostropheus airelensis*, gen. nov.: a review of the systematics of "*Liliensternus*" *airelensis* from the Triassic-Jurassic outcrops of Normandy (France). *J. Vert. Paleont.* **27**, 73-86 (2007).
6. Padian, K., Hutchinson, J. R. & Holtz, T. R. J. Phylogenetic definitions and nomenclature of the major taxonomic categories of the carnivorous Dinosauria (Theropoda). *J. Vert. Paleont.* **19**, 69-80 (1999).
7. Xu, X. *et al.* A Jurassic ceratosaur from China helps to clarify avian digital homologies. *Nature* **459**, 940-944 (2009).
8. Sereno, P. C. A rationale for phylogenetic definitions, with application to the higher-level taxonomy of Dinosauria. *N. Jb. Geol. Paläont., Abh.* **210**, 41-83 (1998).
9. Brusatte, S. L. & Sereno, P. C. Phylogeny of Allosauroidea (Dinosauria: Theropoda): comparative analysis and resolution. *J. Syst. Palaeont.* **6**, 155-182 (2008).
10. Huene, F. v. Bemerkungen zur Systematik und Stammesgeschichte einiger Reptilien. *Z. Induk. Abstammungs- Vererbungslehre* **24**, 209-212 (1920).
11. Huene, F. v. Die fossile Reptil-Ordnung Saurischia, ihre Entwicklung und Geschichte. *Monogr. Geol. Palaeont. (S 1)* **4**, 1-361 (1932).
12. Paul, G. S. *Predatory dinosaurs of the world*. (Simon & Schuster, 1988).
13. Currie, P. J. & Zhao, X.-J. A new carnosaur (Dinosauria, Theropoda) from the Jurassic of Xinjiang, People's Republic of China. *Can. J. Earth Sci.* **30**, 2037-2081 (1993).
14. Evers, S. W., Rauhut, O. W. M., Milner, A. C., McFeeters, B. & Allain, R. A reappraisal of the morphology and systematic position of the theropod dinosaur *Sigilmassasaurus* from the "middle" Cretaceous of Morocco. *PeerJ* **3**, 1323 (2015).
15. Hendrickx, C., Araujo, R. & Mateus, O. The non-avian theropod quadrate I: standardized terminology with an overview of the anatomy and function. *Peerj* **3**, 1245 (2015).
16. Figari, E. G., Scasso, R. A., Cúneo, R. N. & Escapa, I. Estratigrafía y evolución geológica de la Cuenca de Cañadón Asfalto, Provincia del Chubut, Argentina. *Latin Am. J. Sediment. Basin Anal.* **22**, 135-169 (2015).
17. Wang, J. *et al*. Age of Jurassic basal sauropods in Sichuan, China: A reappraisal of basal sauropod evolution. *GSA Bull*. **130**, 1493-1500 (2018).
